# Common Starlings (*Sturnus vulgaris*) increasingly select for grazed areas with increasing distance-to-nest

**DOI:** 10.1371/journal.pone.0182504

**Published:** 2017-08-03

**Authors:** Henning Heldbjerg, Anthony D. Fox, Peder V. Thellesen, Lars Dalby, Peter Sunde

**Affiliations:** 1 Department of Bioscience–Wildlife Ecology, Aarhus University, Kalø, Rønde, Denmark; 2 DOF BirdLife Denmark, Copenhagen, Denmark; 3 Hjortkær, Årre, Denmark; Hungarian Academy of Sciences, HUNGARY

## Abstract

The abundant and widespread Common Starling (*Sturnus vulgaris*) is currently declining across much of Europe due to landscape changes caused by agricultural intensification. The proximate mechanisms causing adverse effects to breeding Starlings are unclear, hampering our ability to implement cost-efficient agri-environmental schemes to restore populations to former levels. This study aimed to show how this central foraging farmland bird uses and selects land cover types in general and how use of foraging habitat changes in relation to distance from the nest. We attached GPS-loggers to 17 breeding Starlings at a Danish dairy cattle farm in 2015 and 2016 and analysed their use of different land cover types as a function of distance intervals from the nest and their relative availability. As expected for a central place forager, Starlings increasingly avoided potential foraging areas with greater distance-to-nest: areas ≥ 500 m were selected > 100 times less frequently than areas within 100 m. On average, Starlings selected the land cover category Grazed most frequently, followed by Short Grass, Bare Ground, Meadow and Winter Crops. Starlings compensated for elevated travel costs by showing increasing habitat selection the further they foraged from the nest. Our results highlight the importance of Grazed foraging habitats close to the nest site of breeding Starlings. The ecological capacity of intensively managed farmlands for insectivorous birds like the Starling is decreasing through conversion of the most strongly selected land cover type (Grazed) to the least selected (Winter Crops) which may be further exacerbated through spatial segregation of foraging and breeding habitats.

## Introduction

In Western and Central Europe, bird populations associated with farmland habitats have been steadily decreasing for three decades (e.g. [1–4]). These long term and large scale population declines of multiple species have been associated with ‘agricultural intensification’ [2,5]. However, the underlying specific, proximate causes hidden beneath such a generic explanation (which may result from multiple causes, even for a single species) often remain poorly understood. For instance, populations may decline or disappear entirely due to: (1) general deterioration in food availability, caused by intensified cultivation, (2) conversion of land cover types providing rich feeding opportunities to other cover types of less or no value and/or (3) increasing homogenisation of the agricultural mosaic (e.g. larger field units), reducing micro-habitat presence and diversity (e.g. nesting and foraging habitats) within an individual’s activity range [6–8]. Although the overall process of agricultural intensification may not be reversible, the adverse effects on biodiversity components (such as species abundance) may be diminished or reversed through agri-environmental schemes or voluntary means. Such beneficial actions should include modest management targets to achieve the highest possible biodiversity benefit in a given landscape context. To achieve the best results, knowledge of a species’ micro habitat requirements, which include the spatial configuration of these elements in relation to each other, are of paramount importance. To understand these requirements, predictive models of habitat suitability based on use by observed individuals relative to habitat availability, so called Resource Selection Functions (RSFs), have increasingly been used [9–11]. Ecological inference from RSFs is based on the premise that selection (i.e. the disproportionate use of a resource relative to its availability) reflects optimal behavioural decisions made by an organism in response to relative habitat quality, since relative differences in selection between habitat units are likely to be approximately proportional to their difference in quality [11–12]. The logic is that if a given habitat type, A, is used x times as often as another habitat type, B, if equally available and all other factors are held equal, then habitat A can be assumed to be approximately x times (the selection ratio or odds ratio of selection) as important as B. This is the case as long as both habitats are used for exactly the same purpose (e.g. foraging). Similarly, by simultaneously modelling several habitat variables, RSFs can estimate partial variation in habitat quality (as a proxy) as a function of individual habitat traits in a given environmental context (all other habitat factors held equal). RSFs can also quantify selection for different land cover types at different distances from the nest. Finally, maps of individual selectivity can be generated from RSFs comprising multiple predictors (e.g. effects of land cover types and distance-to-nest), which in turn can be considered as fairly reliable proxies for the relative importance of the individual area units within the studied subjects’ home range [11–12].

In this study, we present a spatially explicit habitat selection model for foraging Common Starlings (*Sturnus vulgaris*, hereafter Starling) breeding in a Danish farmland landscape. We use recent developments in lightweight GPS logger technology to track foraging Starlings on a traditionally managed dairy cattle farm in southern Jutland in order to determine their habitat use in relation to land cover type (i.e. habitat) availability and distance-to-nest.

The Starling is one of Europe’s most common and geographically widespread farmland birds, occupying a wide range of open habitat types, feeding on invertebrates harvested from the ground and upper soil horizons. As such, it is a perfect model species to study of how ‘common’ insectivorous farmland birds adapt to prevailing food conditions in farmland habitats. The Starling is a short distance migrant and a common and widespread summer visitor to Denmark, where breeding abundance has significantly declined by 2.2% per annum during 1976–2015 [13], similar to the mean annual decline of 1.9% throughout Europe during 1980–2013 [14]. During 1976–2015, Danish farmland has changed considerably through intensification and specialisation, with a major shift from spring sown to autumn sown cereals during the 1980s and 1990s and a reduction in number, an increase in mean size and an increasing concentration of dairy farms in the south and west of the country [15–16]. The extent of grazed grassland has decreased as the area of intensively managed silage grassland and maize cultivation has increased, because dairy cows are now predominantly fed indoors [4]. An earlier national study showed contrasting trends in breeding Starling abundance between Danish regions depending on the regional land use change, particularly in relation to grassland area and intensity of cattle grazing [16]. However, in the absence of data on precise habitat use by Starlings within the farmland mosaic, it is difficult to gather more than correlational support for hypotheses regarding their population declines.

Starlings provisioning offspring become classic central-place foragers, harvesting invertebrate prey and returning to a central place (the nest site) to deliver food to their nestlings. This provides a unique opportunity to determine habitat selection at a landscape scale in relation to availability, and look at the interaction between selectivity and distance. Since prolonging foraging trip duration is both energetically costly and reduces trip frequency, we predict that habitat selection will be greater with increasing distance, increasing load size or energy content to elevate profitability [17]. For these reasons, we deployed GPS loggers on breeding male and female Starlings solely while feeding their nestlings, when habitat choice is critical to their foraging efficiency, and to their reproductive success. Since it is known that breeding Starlings select especially short sward grasslands [18–19], we were curious to see how such habitats (grazed and ungrazed, and others) were selected in the agricultural landscape by individual birds from the same colony in relation to their relative availability.

## Methods

### Study site

The study site was chosen at a colony on a traditional dairy farm with grazing cattle and mixed crops of spring barley, winter wheat, grass and fodder beets, owned by PVT in Hjortkær, near Esbjerg (55°32.4077’N 8°43.6529’E) in the area of southwest Denmark. Starlings in this part of Denmark have shown the least declines in abundance nationally [16] and hence this area was chosen because it was expected to represent birds showing least disturbed behaviour in the country. All studied Starlings bred in 27 nestboxes mounted on farm buildings or surrounding trees within 50 m of each other, 3–4 m above the ground. Breeding success at this colony has been stable since 1971 [20] and occupancy was more or less 100% until c. 10 years ago, since when the proportion has fallen to roughly 60%.

### GPS loggers

We attached battery powered Gypsy 5 GPS loggers (Technosmart Europe srl., Rome) with a total mass (including Teflon harness) of c. 3.2 g (c. 3.5–4% of Starling body mass) and positional accuracy down to 2–4 metres. All loggers were set to record 1 fix/minute during the daylight period and either low frequency (1 fix /hour) or no fixes at night (night positions were not considered in this study), commencing one day after attachment to exclude eventual behavioural effects of being caught and handled. At this frequency of positional logging and a battery capacity of up to 32 hours, we re-caught tagged birds after 3–5 days to retrieve loggers to obtain as much data as possible, extracting data via a cable connection.

### Capture and instrumentation

The capture and instrumenting of Starlings used in this study conforms with the Aarhus University code of practice to ensure responsible research conduct and was carried out with the expressed permission of the Ringing Centre of the Danish Natural History Museum. Breeding Starlings were caught during 5–14 May 2015 (7) and 6–14 May 2016 (10) either in nestboxes or in mist nets nearby while provisioning offspring (see S1 Table). There was very little variation in the clutch size and pairs at the colony breed highly synchronously (Thellesen in print), so the ringing/logging period was chosen to include a comparable group of adult birds with chicks at the age of 4–7 days. Hence, we consider breeding stage and brood size had little effect on individual behaviour. Individual body mass of provisioning Starlings varied by several grams per day, so mass at capture was not incorporated into the models. Starlings were fitted with a logger using a harness of 2 mm Teflon ribbon around each wing, held together by a short 4 mm Teflon ribbon in front of the sternum. Harness straps were either knotted, sewed and glued to the logger on the back of the bird (in 2015) or secured by a metal clipped loop to the logger (in 2016). For two experienced ringers the handling time was 10–20 minutes including ringing, colour ringing and attachment of the logger. Data were extracted from 17 individual first clutch breeding birds (6–32 hours of data per bird; S1 Table). Despite a potential day-to-day variation in prey availability within the c. one week per year the data were sampled, we consider the data derived from all loggers from the same year as samples of the same general conditions of prey availability in relation to habitat availability.

### Starling positions and filtering the data

Each logger provided a data file (.txt) giving information on every fix until the battery runs short of energy. Each fix comprised information on time, position, speed and precision see [21] and S1 Fig.

We filtered the data to focus on habitat use only while foraging and included only precise positions. We excluded all data of birds flying (speed > 0.0) and all data with a precision less than c. 10 m (HDOP > 2.5) to maximize the number of data points assigned to foraging habitat categories. We also excluded all data between 20:00 and 05:30 local time to exclude positions related to movements to and from night roosts. Finally we excluded all data from the habitat categories buildings, gardens, forests and lakes (10.4% of total area within 1 km from the colony) since these were clearly associated with behavioural activities other than feeding (e.g. sleeping, roosting, singing, mating, drinking etc.) and were not relevant in the context of describing foraging behaviour. We only included birds/loggers with more than 50 observations which resulted in 52–382 (mean ± SE: 241 ± 25, S1 Table) positions per Starling.

### Description and coding of land cover types and distance-to-nest categories

All uniform habitats out to a radius of 1 km from the capture site (in excess of the maximum distance from the nest where the Starlings foraged) were described to habitat/crop on maps within field and land parcel units, to define their relative availability to foraging Starlings. Field polygons were retrieved from the common agriculture register (GLR, “Det Generelle Landbrugsregister”) maintained by the Danish AgriFish Agency [22]. For all other habitat categories (meadows, forests, etc.) we used publicly available map layers from the Danish public geographical administration data (GeoDanmark, downloaded 2012, http://download.kortforsyningen.dk). This habitat information was transferred to GIS layers by defining polygons for all registered crops.

**Fig 1:**
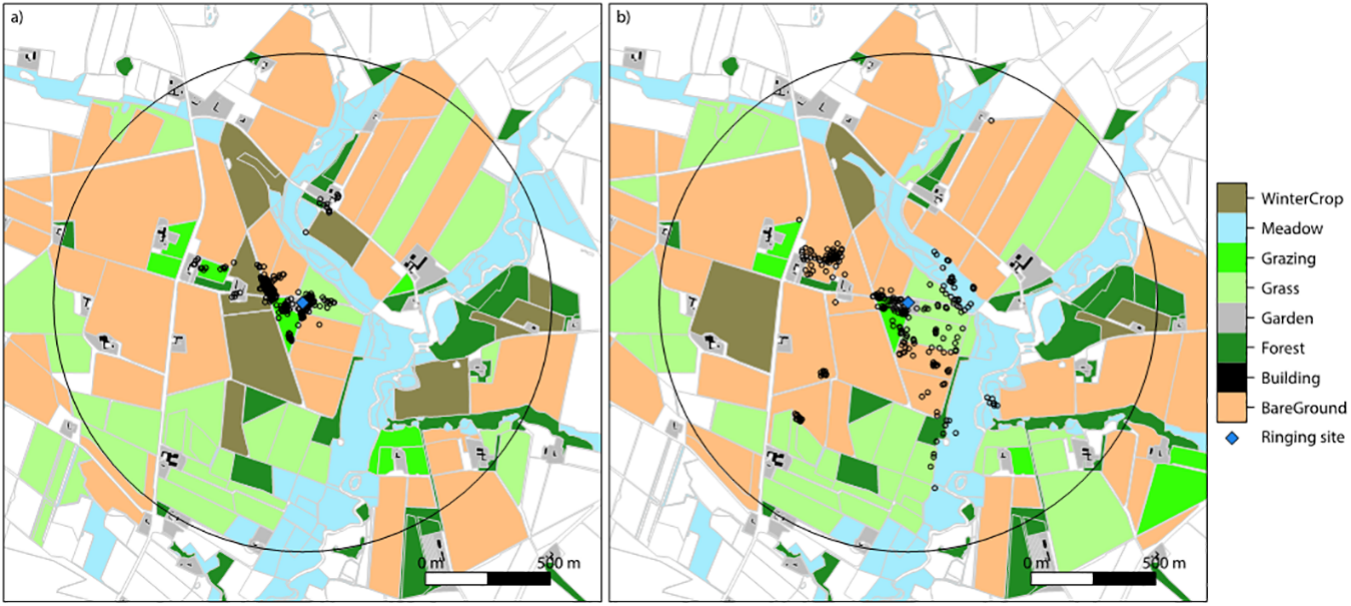
Positional fixes from two different foraging breeding Starlings (*Sturnus vulgaris*). The map shows positions of two different birds (left: S7, 2015 in a) and right: S9a, 2016 in b)) recorded using GPS loggers at a dairy farm in Hjortkær, Jutland, Denmark overlaid on the ringing and nest site (central blue diamond) and the surrounding fields indicating the different crops and the foraging positions of one Starling during c. 24 hours. The categories Building, Garden and Forest are only shown for clarity and were not included in the analysis. The large black circle represents the limit of habitat classification and has a radius of 1000 m (See also S1 Table and S1 Fig in Supplementary Materials for the full set of all mapped individuals).

For the statistical analyses, land cover types were condensed into five predominant crop categories available for all birds: Grazed (grazed grass, but management otherwise is unknown), Short Grass (at the time of the study), Bare Ground (new-sown maize and spring cereals), Winter Crops (autumn-sown cereals and rape) and Meadows (non-grazed/mown grassland) (Fig 1).

Habitat availability was measured as the frequency of each land cover type at different distance-to-nest categories. This was determined by superimposing a 50 m grid overlaid upon the land-use classification map covering the study area out to beyond the maximum observed Starling foraging distance from the farm. The habitats present at each grid intersection (4 ha^-1^, hereafter referred to as relative availability points for habitat sampling, ‘RAPs’) were then used to generate a set of systematic habitat frequencies to describe their relative availability to Starlings in the study area.

For each recorded GPS fix (use) and RAP (availability) we assigned a habitat category and calculated the distance in metres to the colony (as a proxy for the distance to the nest and hereafter ‘distance-to-nest’), using the centre of the farmyard in which the nest box colony was situated as the fixed point for all nests. In order to quantify the mean proportional use of Starlings within the various distance-to-nest distance class intervals, as well as determine their habitat selection relative to these intervals, the distance-to-nest measurements were aggregated into intervals of 100 m (i.e. 0–99 m, 100–199 m, etc.).

### Analyses of distance-to-nest patterns and use of land cover types

For each Starling we calculated the average distance-to-nest and the proportion of GPS-fixes classified within the ten 100 m distance-to-nest intervals. Using individual Starlings as the observational unit, we tested whether the 17 Starlings’ average distance-to-nest varied systematically between males and females and between the two study years using a simple general linear model (PROC GLM in SAS 9.4). Since there was no significant systematic variation, the mean proportion of time spent foraging in different distance-to-nest intervals was then calculated as the mean proportion of GPS fixes from each bird within a given distance interval based on simple normal statistics. As most confidence limits fell between 0 and 1, we considered it justified to base our estimates on arithmetic means rather than back-transformed values of logit-transformed means that would result in slightly low-biased estimates. To illustrate the proportional use of land cover types within each of the ten 100 m distance-to-nest intervals, we pooled data from all GPS-positions from all 17 Starlings and RAPs within each distance-to-nest interval to calculate the relative frequencies of Starling use of each habitat type relative to its availability.

We tested for sex differences in land cover use using a multinomial logistic regression model (GLIMMIX procedure in SAS 9.4 [23]) with the five land cover categories as response variable, a generalised logit link function and multinomial error distribution, sex as fixed effect and Starling ID as random effect. Habitats were equally available to all individuals (with only very minor differences between 2015 and 2016). The sex distribution between habitats was also almost identical in the two study years. Hence, the lack of systematic difference in use of land cover types between males and females could be inferred as a lack of difference in habitat selection between males and females, confirmed in the analysis (see Results). Hence, sex was not considered as factor in the subsequent analyses of habitat selection.

### Analyses of habitat selection

Habitat selection was modelled as differential Starling use of habitats relative to their availability as a RSF, comparing GPS positions (representing use) with availability derived from the combined frequencies from RAPs for each Starling as observation units [9]. We used generalized linear mixed models with a logit link function and binomially distributed error terms (‘logistic regression’) to model the relative probability that an observation would be categorized as GPS-location (use) or as RAPs as functions of cover type and/or distance interval to the nest.

The data set consisted of all use (GPS-positions) and availability locations within the five selected land cover categories within 999 m from the colony centre for each of 17 Starlings (i.e. for each Starling a set of RAPs were entered with its ID annotated). To account for different ratios of GPS-fixes and RAPs between individuals, Starling ID was entered as a random factor. To adjust for variance inflation due to individual variation in habitat selection (which may appear as a simple result of serial dependency of consecutively recorded GPS-locations in the same field block unit), as the random effects we included interaction term(s) between Starling ID and all habitat variables entered as fixed effects [24]. All models were run in the GLIMMIX procedure in SAS 9.4 with denominator degrees of freedom estimated using the Satterthwaites approximation method [23].

As follows from RSF theory, the predicted probability provided by a RSF of an observation being a GPS-location as opposed to a RAP observation is uninformative (because the numbers of GPS-fixes and RAPs were arbitrarily chosen). However, the logistic regression coefficients describe the relative log-transformed differences in preference (which is equivalent to the relative difference in use assuming availability is constant) between habitat types when all other influencing factors were held equal in the model [9]. In the present analysis, selection for land cover types was expressed in relation to the land use category Grazed (i.e. how often the other land cover types were used relative to Grazed if equally available) and selection for distance-to-nest intervals as being relative to the 0–99 m interval.

RSFs were constructed for (i) land cover types (five categories) in isolation (i.e. ignoring variation in distance-to-nest), (ii) distance-to-nest intervals (100 m categories) in isolation (ignoring variation in composition of land cover types) and (iii) both variables combined as main effects.

Because some models failed to converge due to unbalanced data amongst individuals (e.g. because of missing observations within certain habitat categories) it was not possible to construct models that incorporated selection for land cover types as function of varying distance-to-nests (interaction terms between distance-to-nest and land cover types). Instead, selection for land cover types at different distance zones from the nest were estimated from separate RSFs constructed for sub-divisions of the data set at 0–199 m, 200–399 m and 400–999 m from the nest. To achieve model convergence, the RSF analysis from the furthest distance-to-nest zone (400–999 m) was restricted to seven Starlings with > 20 GPS locations within this interval (Loggers S2, S3, S5, S8, S9, S9a, S10, S1 Table). Also, the category Winter Crops was excluded from the analysis since no Starling was ever observed in this land cover type > 399 m from the nest. Selection coefficients (SC) for Winter Crops relative to Grazed 400–999 m from the nest were approximated for each individual Starling using the method of [25]. This calculates SC_WC-G_ = ln(U_WC_/A_WC_)–ln(U_GR_/A_GR_)], where U_WC_/A_WC_ and U_GR_/A_GR_ are the proportions of GPS-fixes (used = U) divided by the proportion of RAP availability points (A) from the Starling found in Winter Crops (WC) and Grazed (GR), respectively. In this case, all 0-values (i.e. no observations) of either U or A were replaced by a value equal to 0.5 count observation (so, for example if 0 out of 24 GPS-locations were found in Winter Crops, U_WC_ = 0.5/24 = 0.021). With normal statistics, a mean SC_WC-G_, SE and 95%CI was calculated for each of the six Starlings that used Grazed at least once >399 m from the nest.

Pairwise differences in selection coefficients were estimated from different models as ΔB = B_1_ –B_2_ (where B_1_ and B_2_ are estimate 1 and 2). These were tested on the basis of simple t-statistics: t_(df1+df2)_ = (B_1_-B_2_)/([SE_B1_]^2^+[SE_B2_]^2^)^0.5^, where df_1_ and df_2_ are the degrees of freedom of estimates 1 and 2, and SE_B1_ and SE_B2_ are the SEs of estimates 1 and 2. This method was used to test for differences in selection coefficient estimates of land cover types from a RSF that had land cover types as the only fixed effect and a RSF that incorporated both land cover types and distance-to-nest interval as fixed effects. Similarly, the method was used to test for differences in selection coefficients of distance-to-nest intervals from a RSF that consisted of distance-to-nest intervals as the only fixed effects and a RSF incorporating land cover types and distance-to-nest interval as fixed effects.

## Results

### Use and selection of distance-to-nest intervals

**Fig 2:**
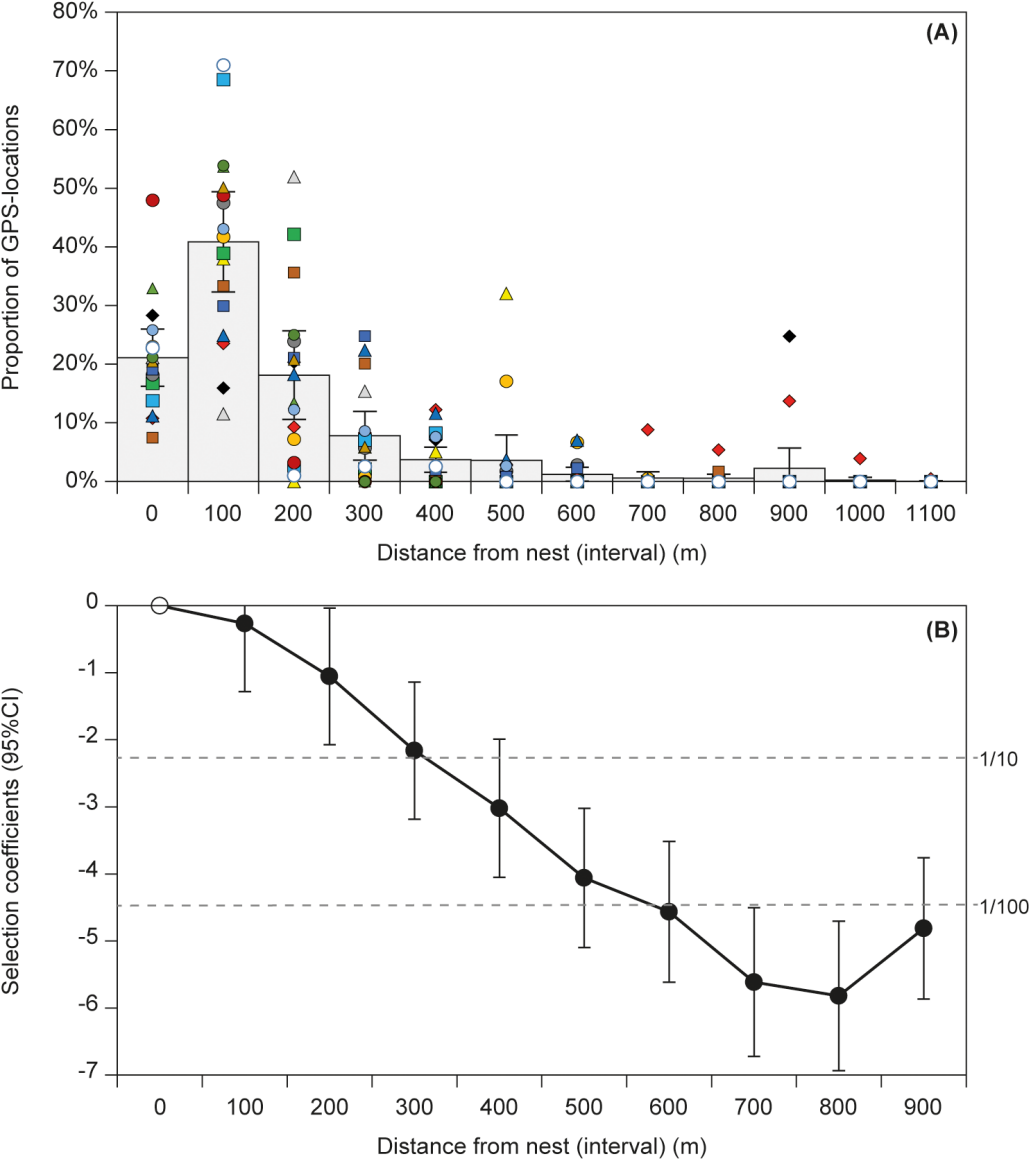
Foraging distance for breeding Starlings. (A) Proportion of GPS-locations of 17 foraging Starlings at different distance intervals from the nest, the different symbols indicate different birds. (B) Selection coefficients of distance intervals relative to the selection in the nearest interval (0–99 m) from RSF that also accounts for selection of land cover types. The anti-log of the coefficients indicate the approximate odds ratio by which a distance category is used relative to availability compared to 0–99 m from the nest (horizontal stippled lines indicate odds ratios of 1:10 and 1:100 as a guide).

The 17 Starlings showed individual differences in activity with distance-to-nest intervals (Fig 2A), but on average 21% (95% CI: 16–26%) of their locations fell within 99 m of the nest, 41% (32–49) within 100–199 m, 18% (11–26) within 200–299 m, 8% (4–12) within 300–399 m and 12% (4–20) >399 m from the nest. Mean activity distances (observations within land cover types included in the RSFs) were similar for males and females (240 vs. 213 m, *F*_1,16_ = 0.45, *P* = 0.5) but tended to be somewhat (although not statistically significantly) greater in 2015 than 2016 (265 vs. 188 m *F*_1,16_ = 3.50, *P* = 0.08).

Predictions for selection of distance-to-nest distance (based on 100 m-intervals) were similar for the RSF that included land cover types and distance categories and the RFS that only included distance-to-nest (Table 1). Areas within the first 199 m were selected most strongly and equally, after which distance intervals were selected increasingly less frequently relative to the use of the area within 0–99 m of the nest (Fig 2B; overall test for the ten distance intervals being equally selected: *F*_9,142_ = 31.8, *P* < 0.0001).

**Table 1:** Model parameters of RSFs for habitat selection.

		Model = Cover type + distance					Model = Distance only					Model = Cover type only					Difference in selection coefficients				
*Fixed effects*:		B	SE(b)	df	*t*	*P*	B	SE(b)	df	*t*	*P*	B	SE(b)	df	*t*	*P*	ΔB	SE(ΔB)	df	*t*	*P*
cover type	Grazing (ref.)	0.00	.	.	.	.						0.00	.	.	.	.	.	.	.	.	.
	Short Grass	-0.94	0.33	59.1	-2.89	0.005						-1.56	0.32	50.0	-4.9	<0.0001	-0.62	0.46	109	-1.35	0.18
	Bare Ground	-1.83	0.32	58.4	-5.64	<0.0001						-2.10	0.32	49.7	-6.6	<0.0001	-0.27	0.46	108	-0.60	0.55
	Meadow	-2.55	0.33	65.2	-7.66	<0.0001						-3.01	0.33	53.8	-9.2	<0.0001	-0.46	0.47	119	-0.98	0.33
	Winter Crop	-3.87	0.43	107	-8.97	<0.0001						-4.48	0.37	91.6	-12	<0.0001	-0.61	0.57	199	-1.06	0.29
	Overall effect of cover type: F_4,77.7_ = 54.10, P<0.0001											F_4, 56.8_ = 42.4, P<0.0001									
Distance	0–99 m (ref)	0.00	.	.	.	.	0.00	.	.	.	.						.	.	.	.	
	100–199 m	-0.27	0.51	119	-0.52	0.604	-0.57	0.56	126.3	-1.02	ns						-0.31	0.76	245	-0.40	0.69
	200–299 m	-1.05	0.51	120	-2.04	0.043	-1.95	0.56	126.8	-3.47	0.0007						-0.90	0.76	247	-1.18	0.24
	300–399 m	-2.16	0.52	123	-4.18	<0.0001	-2.96	0.56	128.2	-5.25	<0.0001						-0.80	0.76	251	-1.05	0.29
	400–499 m	-3.02	0.52	126	-5.81	<0.0001	-3.55	0.57	130.9	-6.27	<0.0001						-0.53	0.77	257	-0.69	0.49
	500–599 m	-4.06	0.52	130	-7.74	<0.0001	-3.98	0.57	131.3	-7.01	<0.0001						0.08	0.77	261	0.11	0.92
	600–699 m	-4.57	0.53	136	-8.61	<0.0001	-4.99	0.58	139	-8.67	<0.0001						-0.42	0.78	275	-0.54	0.59
	700–799 m	-5.61	0.56	172	-9.99	<0.0001	-6.13	0.60	168.9	-10.14	<0.0001						-0.51	0.83	340	-0.62	0.53
	800–899 m	-5.82	0.56	175	-10.3	<0.0001	-6.34	0.61	174.9	-10.41	<0.0001						-0.52	0.83	350	-0.63	0.53
	900–999 m	-4.81	0.53	138	-9.03	<0.0001	-5.28	0.58	142.6	-9.12	<0.0001										
	Overall effect of distance category: *F*_9,142_ = 31.8, *P* <0.0001						*F*_9,144.5_ = 28.1, *P* <0.0001														
*Random effects (covariance parameters)*																					
Starling ID		0.32	0.26	∞	1.24	0.22	0.45	0.25	∞	1.77	0.11	0.10	0.11	∞	0.95	0.34					
Starling ID*Distance		2.13	0.29	∞	7.36	<0.0001	2.60	0.34	∞	7.70	<0.0001	.	.		.	.					
Starling ID*Cover type		0.78	0.16	∞	4.75	<0.0001	.	.				0.82	0.17	∞	4.74	<0.0001					

Model parameters and overall statistical significance (type-III effects) of variables of Resource Selection Functions for habitat selection by GPS-tagged Starlings 0–999 m from the nest site.

### Use and selection of land cover types

**Fig 3:**
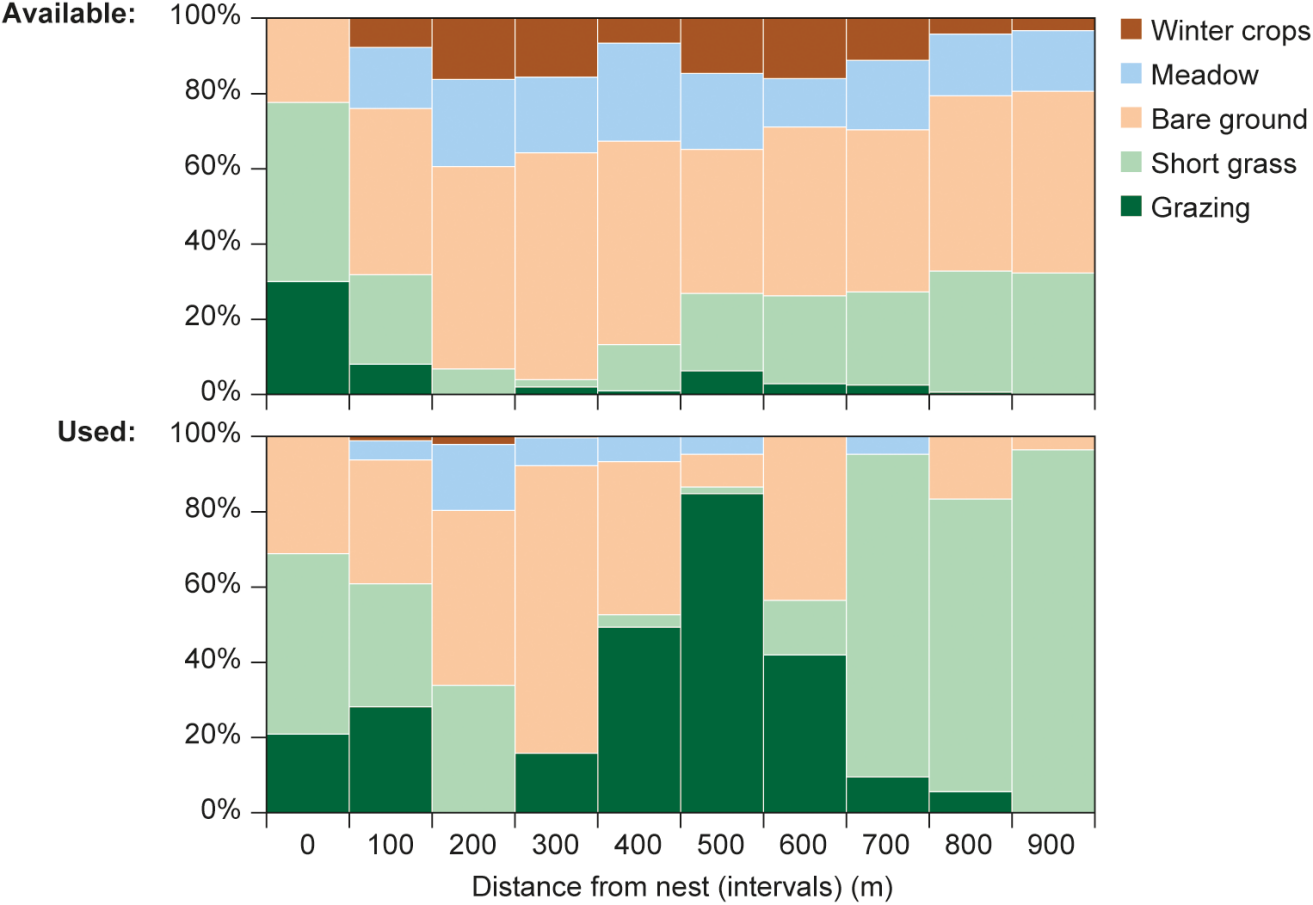
Availability and use of land cover types by 17 GPS-tagged Starlings. The availability and use of land cover types is shown at different distance intervals from the nest (all fixes in a given distance interval pooled across individuals).

Of all 4037 GPS fixes used in the analysis, 37% were on Bare Ground, 32% on Short Grass, 24% on Grazed, 6% on Meadows and 0.8% on Winter Crops with no difference in proportional land cover use between the sexes (*F*_4,23.1_ = 0.71, *P* = 0.59). Use, as well as availability of land cover types, varied with distance-to-nest (Fig 3).

**Fig 4:**
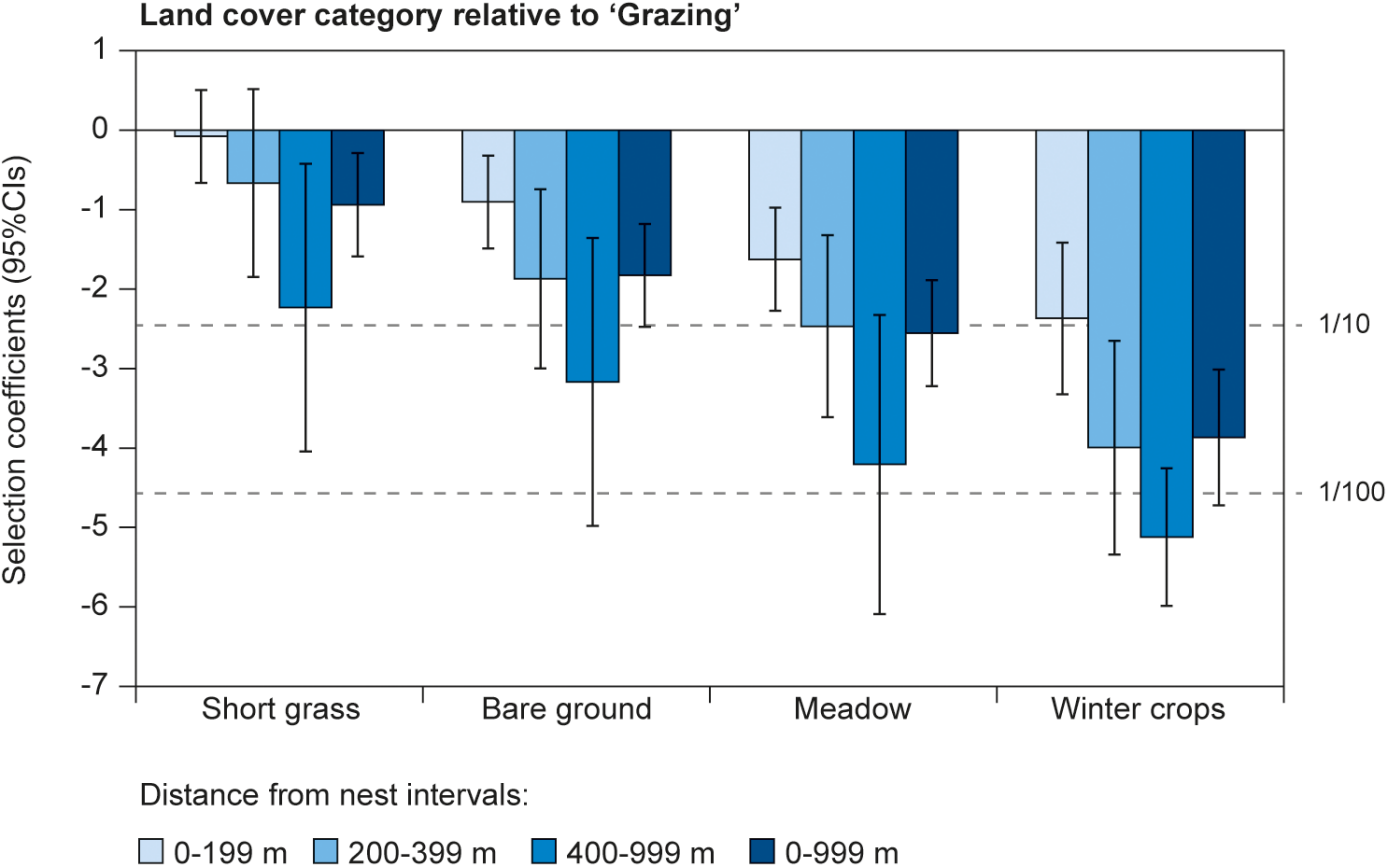
Selection for land cover types. Selection for land cover types is shown relative to “Grazing” by 17 GPS-tagged Starlings at different distance-to-nest intervals (within which selection for 100 m distance intervals are accounted for unless otherwise stated). The anti-log of the coefficients gives the approximate odds ratio to the frequency by which a land cover category is used relative to grazed areas if availability is the same (horizontal stippled lines indicate odds ratios of 1:10 and 1:100 as a guide).

According to both RSFs (i.e. those which only included land cover types and those which also accounted for selection within distance-to-nest intervals), at 0–999 m from the nest, Starlings strongly selected between land cover types (Table 1). The two modelling alternatives resulted in similar selection coefficients, which did not differ significantly (Table 1). Starlings selected the land cover category Grazed significantly more than all other land cover types, followed by Short Grass (selection ratio to Grazed = 1:2.6), Bare Ground (1:6) and Meadow (1:13), with Winter Crops by far the least selected cover type (1:48, Table 1, Fig 4). Hence, within cultivated fields, Bare Ground (spring sown crops) was selected eight times (95% CI: 3–18 times) more than Winter Crops (Table 1).

When RSFs were constructed for different distance-to-nest intervals, selection for land cover types varied with increasing distance to nest, as Grazed was selected significantly more than other cover types 400-999m from the nest compared to within the first 199 m from the nest (Table 2, Fig 4). Hence, while Short Grass was selected approximately equally to Grazed at 0–199 and 200–399 m from the nest, Grazed was selected significantly over Short Grass at a ratio of 9:1 further away than 399 m from the nest.

**Table 2:** Selection of land cover types.

Distance intervals	Selection coefficients for land cover types relative to "Grazed"																				Test for overall selection		
	Short Grass					Bare Ground					Meadow					Winter Crops							
	B	SE(b)	df	*t*	*P*	B	SE(b)	df	*t*	*P*	B	SE(b)	df	*t*	*P*	B	SE(b)	df	*t*	*P*	*F*	df	*P*
A: 0–199 m	-0.08	0.29	38.9	-0.27	0.79	-0.90	0.29	38.7	-3.13	0.003	-1.62	0.32	59.5	-5.01	<0.0001	-2.37	0.48	93.1	-4.93	<0.0001	12.7	4,52	<0.0001
B: 200–399 m	-0.67	0.59	83.9	-1.12	0.27	-1.87	0.57	71.7	-3.31	0.002	-2.47	0.57	76.4	-4.29	<0.0001	-4.00	0.68	88.0	-5.90	<0.0001	13.9	4,63.5	<0.0001
C: 400–999 m	-2.23	0.87	21.3	-2.56	0.02	-3.17	0.87	21.3	-3.64	0.002	-4.21	0.92	26.1	-4.59	<0.0001	-5.12	0.43	5.0	-11.9	<0.0001	7.92	3,23	0.0008
difference: A-B	0.59	0.66	122.8	0.89	0.38	0.97	0.63	110.3	1.52	0.13	0.84	0.66	135.9	1.28	0.20	1.63	0.83	181	1.96	0.051			
difference: B-C	1.57	1.05	105.2	1.49	0.14	1.30	1.04	93.0	1.25	0.21	1.74	1.08	102.4	1.61	0.11	1.12	0.80	93.0	1.40	0.16			
difference: A-C	2.16	0.92	60.2	2.35	0.02	2.27	0.92	60.0	2.47	0.002	2.58	0.97	85.6	2.66	0.009	2.75	0.64	98.1	4.27	<0.0001			

Coefficients for selection of land cover types relative to “Grazed” and F-statistics for test for overall selection of land cover types for analyses split on different distance-to-nest intervals from the nest for GPS-tagged Starlings.

## Discussion

The deployment of GPS logger units on Starlings in this study provided data with high spatial accuracy on space use and habitat selection of foraging adult breeding birds during the critical period when they seek to maximise food provisioning. The results clearly showed Starlings foraged at distances more than 500 m from the nest more than 100 times less frequently than within 100 m and selected Grazed grassland over all other habitat category types in the farmland landscape. These novel results provide three key implications for agri-environmental management schemes.

Firstly, this study quantified the differences in use by foraging Starlings between common available crops in a Danish farmland landscape and illustrated how some crops were clearly avoided by Starlings foraging to provision their offspring. The strong selection for Grazed areas confirmed the importance of grazing livestock (in this case cattle) for maintaining short-grazed high quality foraging habitats for Starlings. Grazed areas were selected 2.6 times more than ungrazed (at the time of the study) Short Grass and six times more than Bare Ground. It seems likely that food items should be equally accessible in these crops (relative to their density) for foraging Starlings, implying that Grazed habitats host a higher density of available prey compared to cut Short Grass and Bare Ground (i.e. ploughed cultivated fields for spring crops). Grazed grassland was selected 13 and 48 times more than Meadow and Winter Crops, while Bare Ground (i.e. new sown spring crops) was selected by a factor of 8 over Winter Crops, indicating the relative profitability of foraging between these habitats. If we assume that microhabitat selection coefficients from RSFs reflect differences in habitat quality, then the behaviour of the tagged breeding Starlings supports the hypothesis that long term population declines in Starling populations in Denmark and other western European countries are causally linked to the transformation of actively grazed pastures to managed silage swards and cultivated crops. It therefore follows that the conservation of actively grazed areas is one of the main key actions likely to guard against further loss of foraging habitats for Starlings and other ground feeding insectivorous farmland birds in order to safeguard these populations from further declines in the future [26].

Secondly, the shape of the activity and selection patterns for distance-to-nest intervals indicate that areas further than 200 m from the nest increasingly lose foraging value with increasing distance, a pattern that undoubtedly relates to increasing travelling costs between nest and foraging sites. In practice, this means that disconnection of breeding sites (i.e. safe nesting cavities in buildings or holes in trees) from foraging habitats at scales beyond a few hundred metres is likely to reduce the quality of potential foraging habitats because increased commuting costs reduce foraging time and elevate flight energy expenditure [27].

Thirdly, the selection increased with foraging distance. Starlings became increasingly selective in their habitat choice the longer they flew, i.e. the more they invested in the foraging trip. Starlings were thus able to compensate to some extent for more widely distributed resources in the landscape by concentrating on exploiting the most profitable patches. Starlings breeding in modern agricultural landscapes (i.e. with large fields and long commuting distances between nest and foraging sites) will profit more by access to limited patches of very high foraging value (e.g. cattle grazed pastures) than larger areas of modest foraging quality (e.g. grass or open land), which fail to balance the energetic costs of commuting to and from the nest site.

### Land cover types and the significance of habitat quality

Starlings showed strong preferences for Grazed over Bare Ground and Short Grass, avoiding Meadows and Winter-crops. These significant differences between habitat types likely mirror food accessibility. Starlings mainly prey on larvae of crane flies (*Tipulidae*) but also those of butterflies, moths and beetles [28]. We were unable to study prey availability in the different crops at our study site but [29] found greater prey abundance in pasture and other permanent grass than in cereal fields in South Sweden and we see no reason why this pattern should be different at our study site. The abundance and density of invertebrates is also lower in intensive grassland monocultures than in extensively managed grassland [30]. As well as density, prey availability is likely affected by vegetation height [19,31–32], because the least preferred tall Winter Crops such as rape or winter wheat reach vegetation heights of c. 150 cm and 40–50 cm, respectively, during the Starling breeding season. In contrast, spring sown crops such as maize (common in the area), fodder beet (rare) and spring sown cereals (mainly barley, which is relatively common) show such late season growth that extensive Bare Ground remains between growing plants during the Starling breeding season, providing additional foraging opportunities for Starlings. Grazed grassland seems to provide the most optimal combination of high prey density and easy access to prey.

At the study site, Grazed grass was available in abundance immediately adjacent to the nest colony, which could explain the consistently high breeding density of Starlings here. The local composition of crops has remained largely unchanged during 1971–2016, during which time there has been no significant change in the mean production of nestlings produced per pair [20]. In order to obtain a better understanding of what influences density and accessibility of the prey, we would also need to consider the variation in mechanical and chemical treatment of the fields as well as determining the effect the presence of grazing cattle has *per se*, but such investigations were beyond the scope of this study.

### Activity distance and the significance of resource dispersion

Ninety-two percent of foraging positions recorded from 17 Starlings of both sexes in two years were within 500 m of the nest, indicating the importance of foraging areas close to nest sites. Wiersma *et al*. [33] showed that daily flight times increased 4-fold with a 3-fold decline in food availability to caged Starlings, increasing daily energy expenditure by 43%. Starlings make up to 250 feeding roundtrips per day [28], so an increase from 100 m to 200 m will increase the total daily travelled distance from 50 km to 100 km. At a mean flight speed of 10 m per second (close to the optimal flight speed at minimum metabolic power of 9.4 W [34]) this would increase daily energy expenditure from 47 kJ to 94 kJ. Starling nestlings consume c. 40 g fresh food per day [35] or c. 160 *Tipula* larvae (mean wet weight c. 0.25 g and an energy content of c 4 kJ/g; [28, 35]); corresponding to a daily energy demand of c. 160 kJ per nestling. In this way, adding an additional 100 m to the foraging distance equates to c. 30% of the daily energy requirements of one nestling. Thus, extending foraging trips will adversely affect breeding success, reflected in the negative relationship between nestling feeding frequency and adult foraging distance [18], which may reduce nestling survival.

### Increased selection with increasing foraging distance

As far as we are aware, few avian studies (e.g. Ring-billed Gulls *Larus delawarensis* [17] and Cinereous vulture *Aegypius monachus* [36]) have demonstrated an increase in habitat selection with distance-to-nest. In this study, we analyse the importance of distance and habitats, and combine them to analyse the relative importance of the different habitat types at different distances from the Starlings nesting site. The significance of Grazed grass for foraging Starlings in the agricultural landscape was further supported by the fact that the selection for this habitat type became more pronounced with increasing distance.

This result conforms to expectations, since the area within each additional 100 m wide circular distance bands away from the nest is increasing, thus providing a greater number of foraging opportunities if and when the Starling invests in longer foraging trips. However, this requires greater habitat selection to compensate for the increasing energetic costs associated with flying longer distances.

Nesting Starlings are classic central place foragers (as shown by aviary studies, e.g. [37] and in the field, e.g. [28]) and our new approach clearly demonstrated increasing habitat selection with distance-to-nest, confirming results from radio-transmitter studies [18]. It seems likely that this is the case for many other species for which such patterns have yet to be demonstrated. Improving telemetry technology offers exciting opportunities to improve our understanding of animal–habitat relationships at finer scales by incorporating interactions to the distance components of RSFs [38].

### The ‘Starling landscape’ and conservation implications

The Starling is highly dependent on two important landscape elements, 1) nest site and 2) foraging areas, as is the case for many other species. Our results show that both resources must be available in close proximity within the ‘Starling landscape’. Within Denmark, the extent of actively grazed grasslands has fallen in recent years as more cattle remain indoors throughout the year [16]. Given these trends and the propensity of Starlings nesting on adjacent farms to commute 500–700 m to forage on selected habitat adjacent to the study farm, it is easy to understand how loss of grazed grassland may have contributed to the observed Starling population declines. Such changes in the agricultural landscape have occurred nationally, as well as at a Western European scale, which may contribute to explaining the differences between declining trends here compared to stable and increasing trends in Eastern Europe [39]. The decline in grazed areas is also known to have an important effect on the populations of Little Owl (*Athene noctua*), another declining species that hunts insect prey in open farmland [40–41].

Summer Starling densities correlated with numbers of grazing cattle and changes in regional Starling breeding abundance correlate with changes in grazing intensity across Denmark [16]. However, even in parts of Denmark with very few cattle, Starlings can persist where a single local farm retains grazing cattle (own observations). Despite a farmland ‘ocean’ of unsuitable habitats within the agricultural mosaic, Starlings can persist if they can find ‘islands’ of nesting and foraging areas in sufficiently close proximity to provide safety from predators and sufficient food to provision nestlings to fledging. Such a habitat matrix is more frequent in western Denmark (where the more dominant dairy farming provides grazed grassland), where Starling densities remain highest and declines have been of least magnitude. In the arable dominated farmland in eastern Denmark, with little or no preferred Grazed or Grassland, the ‘Starling landscape’ is far more restricted and commuting distances are extended to relatively few distant ‘islands’ in far greater ‘oceans’ of unsuitable habitat for the Starling. Smith & Bruun [42] found that both breeding density and production of Starling young per nest was positively related to the availability of pasture close to the breeding colony.

Away from the study farm, loss of actively grazed grassland has presumably caused Starlings nesting in traditionally suitable areas to forage further and further from suitable nesting habitat, to a point where it is no longer energetically profitable to provision offspring at such long distance. This potentially supports the contention that the reduction of pasture in modern agricultural landscape may explain the declining Starling population, as cited by [42]. Elevating Starling breeding abundance can probably better be facilitated by establishing ‘islands’ of high quality Starling habitat across the farming landscape rather than by large areas of modest quality. Future studies should focus on the relationship between provisioning flight distance, foraging profitability and habitat selection in other farmland mosaics, where the distance and availability of habitats contrast the more favourable ones studied here and should also include urban areas and open woodland areas where Starling breed and also show declining trends. Comparisons should also be sought at larger spatial scales. For instance, comparing other parts of Denmark or other countries with less favourable agricultural landscape mosaics for Starlings with central or eastern parts of Europe, where locally breeding Starling numbers are either stable or increasing. The GPS-logger technology provides valuable information on for instance flight distances and home-range sizes e.g. [43] and we have here shown the value of deploying such devices on a species as small as a Starling for the first time, which illustrates the potential for similar studies on a larger number of species.

## Supporting information

S1 Fig Maps of the ringing site and the surrounding fields indicating the different crops and the foraging positions of 17 different tagged Starlings tracked during May 2015 and 2016. (PDF)

S1 File Data set used for analyses of use and selection of habitat categories of GPS-tagged Starlings. (XLSX)

S1 Table Information about the 17 loggers/Starlings showing year, logger, sex, start and length of each logger period, number of foraging positions and registered mean and max distance. (DOCX)
