# Supplementary material for: Common Starlings (Sturnus vulgaris) increasingly select for grazed areas with increasing distance-to-nest
Source: PLoS One. 2017 Aug 3;12(8):e0182504. doi: 10.1371/journal.pone.0182504 (PMC5542446; doi:10.1371/journal.pone.0182504)
Supplement: S1 Fig — (PDF) [file pone.0182504.s001.pdf]

Supporting information for the paper: **Common Starlings (*Sturnus vulgaris*) increasingly selects for grazed areas with increasing distance-to-nest**

Henning Heldbjerg, Anthony D. Fox, Peder V. Thellesen, Lars Dalby & Peter Sunde.

Plos One (2017)

This document contains maps of the ringing site and the surrounding fields indicating the different crops and the foraging positions of 17 different tagged Starlings tracked during May 2015 and 2016.

S1−2016

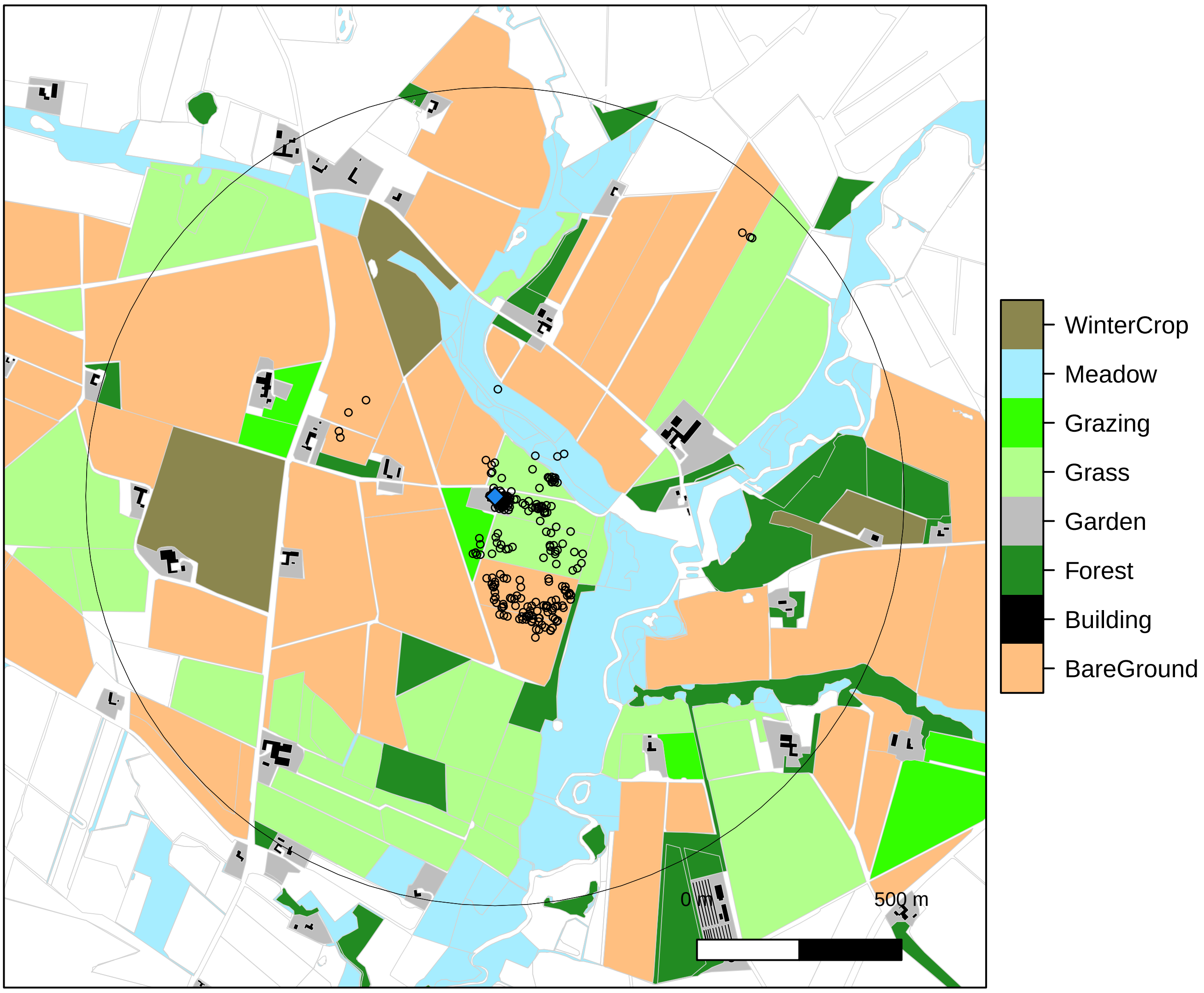
0 m
500 m
WinterCrop
Meadow
Grazing
Grass
Garden
Forest
Building
BareGround

S10−2015

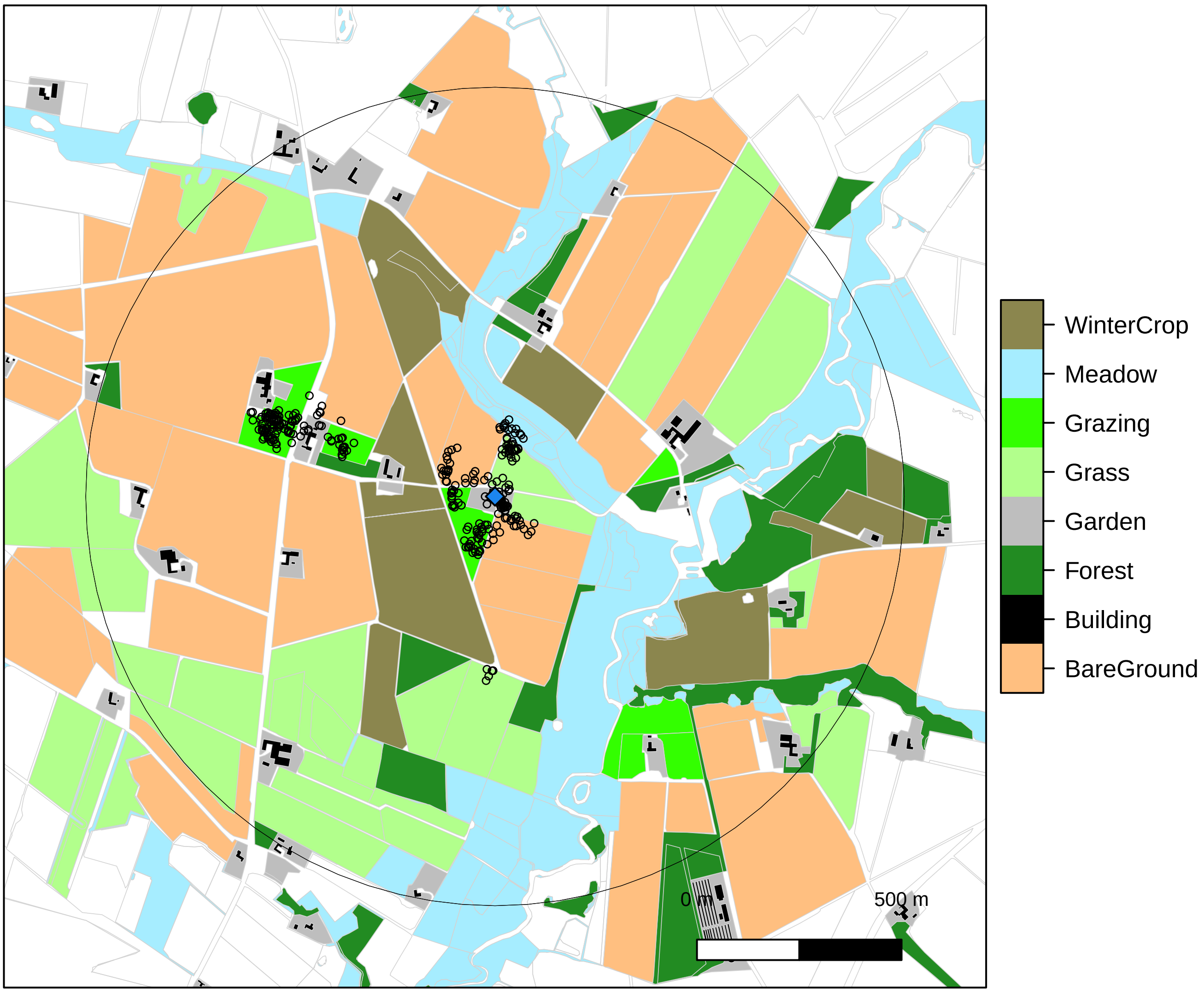
0 m
500 m
WinterCrop
Meadow
Grazing
Grass
Garden
Forest
Building
BareGround

S11−2016

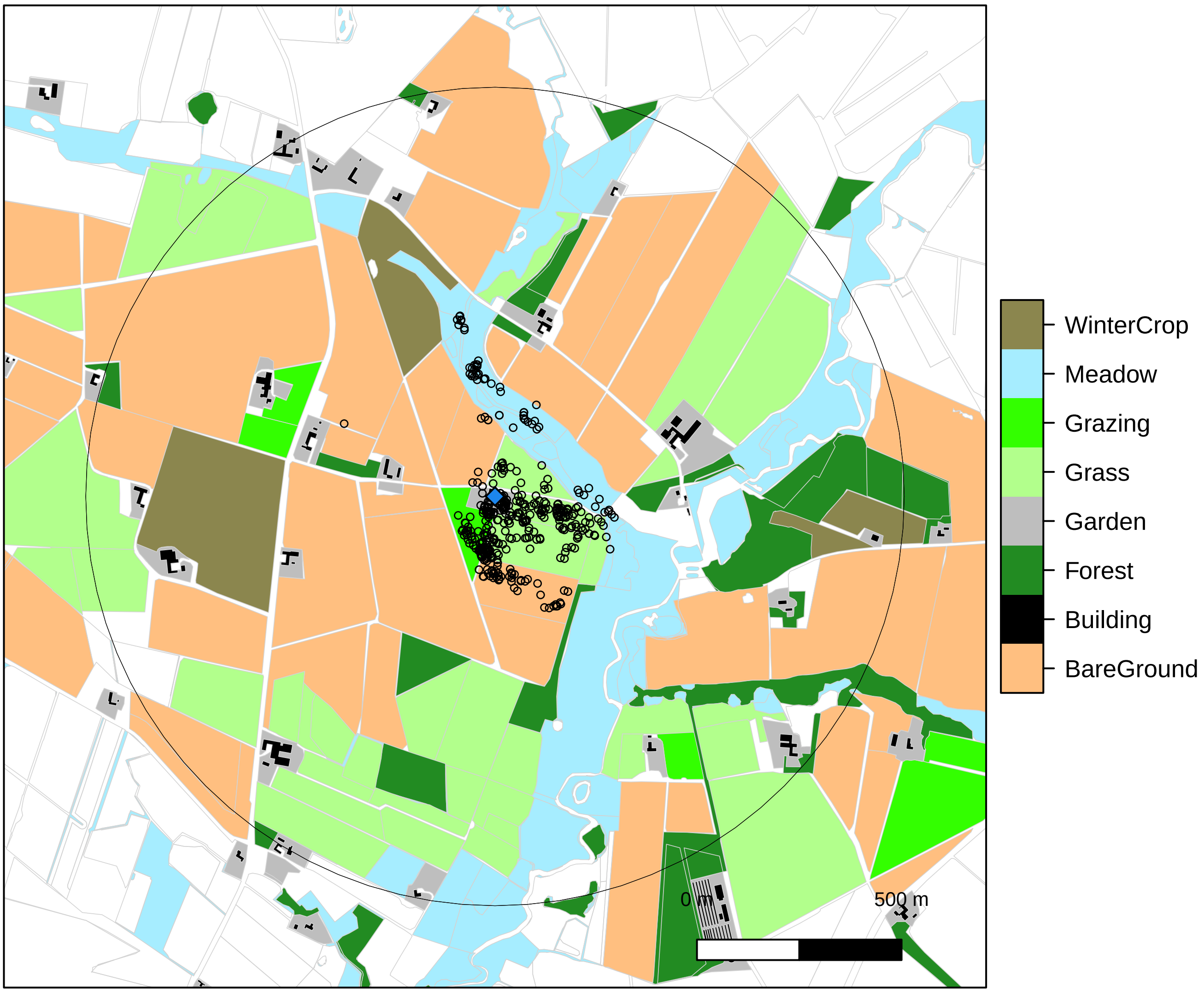

0 m
500 m
WinterCrop
Meadow
Grazing
Grass
Garden
Forest
Building
BareGround

S12−2016

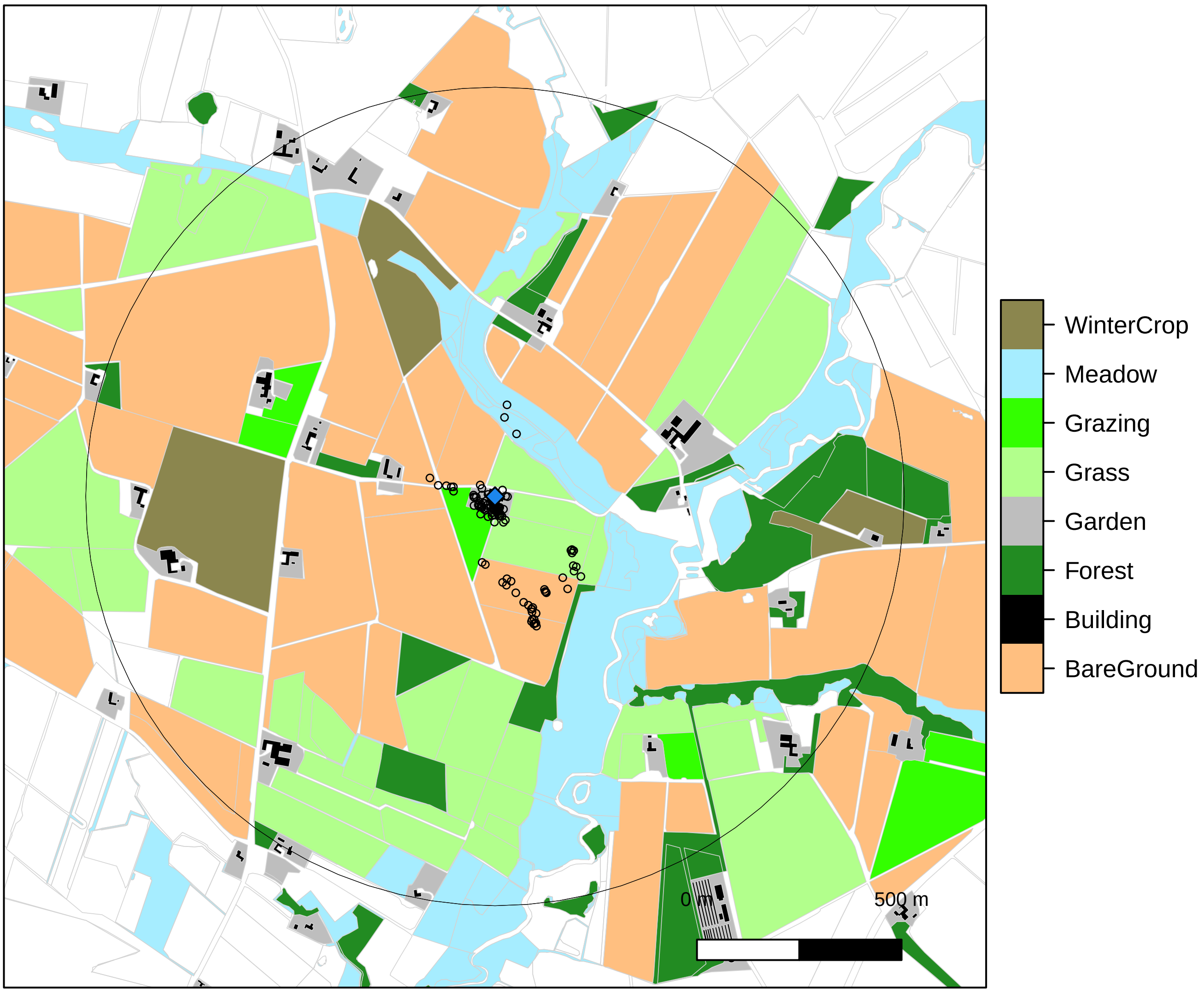

WinterCrop
Meadow
Grazing
Grass
Garden
Forest
Building
BareGround
0 m
500 m

S13−2016

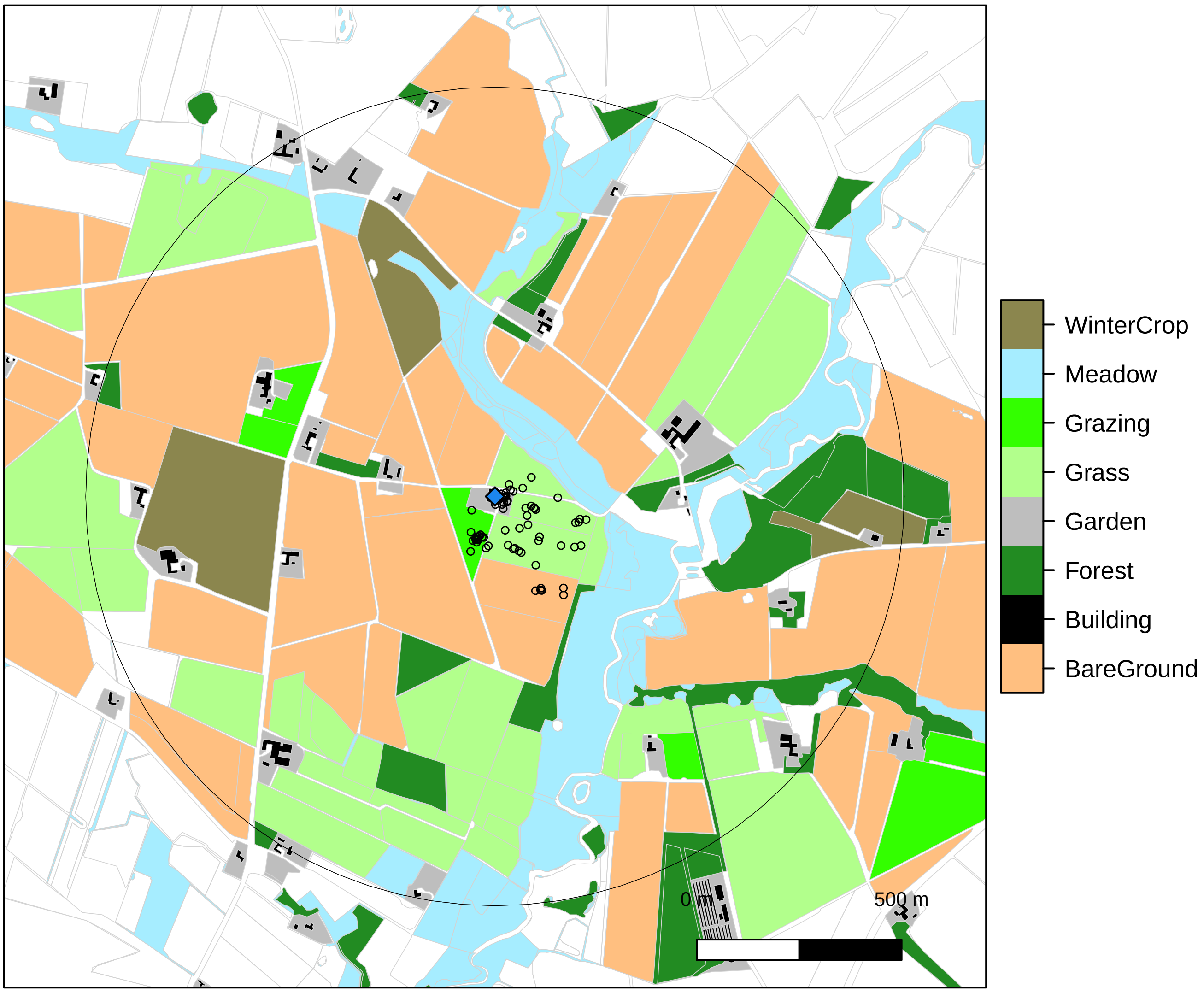

0 m
500 m
WinterCrop
Meadow
Grazing
Grass
Garden
Forest
Building
BareGround

S14−2016

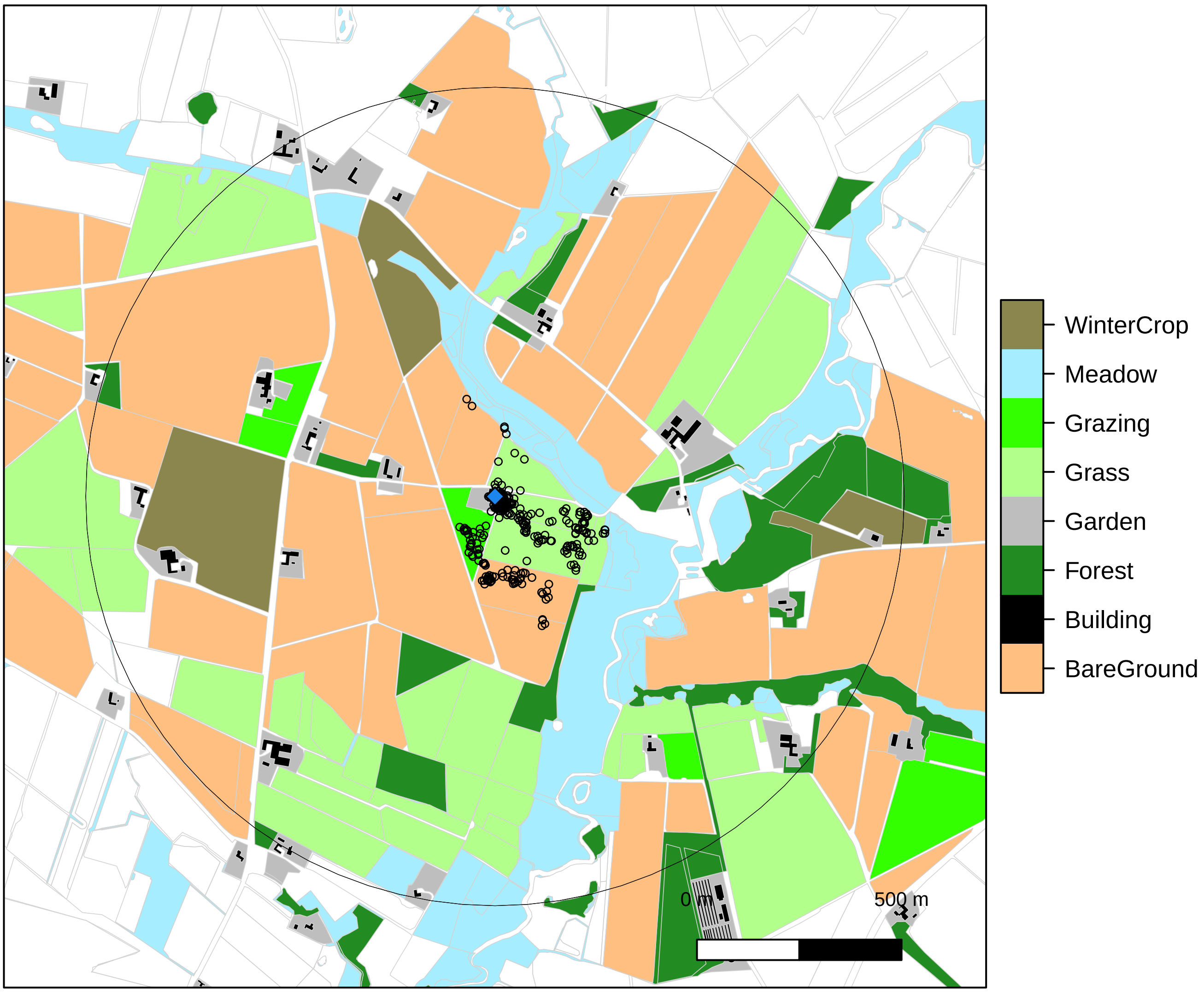
WinterCrop
Meadow
Grazing
Grass
Garden
Forest
Building
BareGround
0 m
500 m

S17−2016

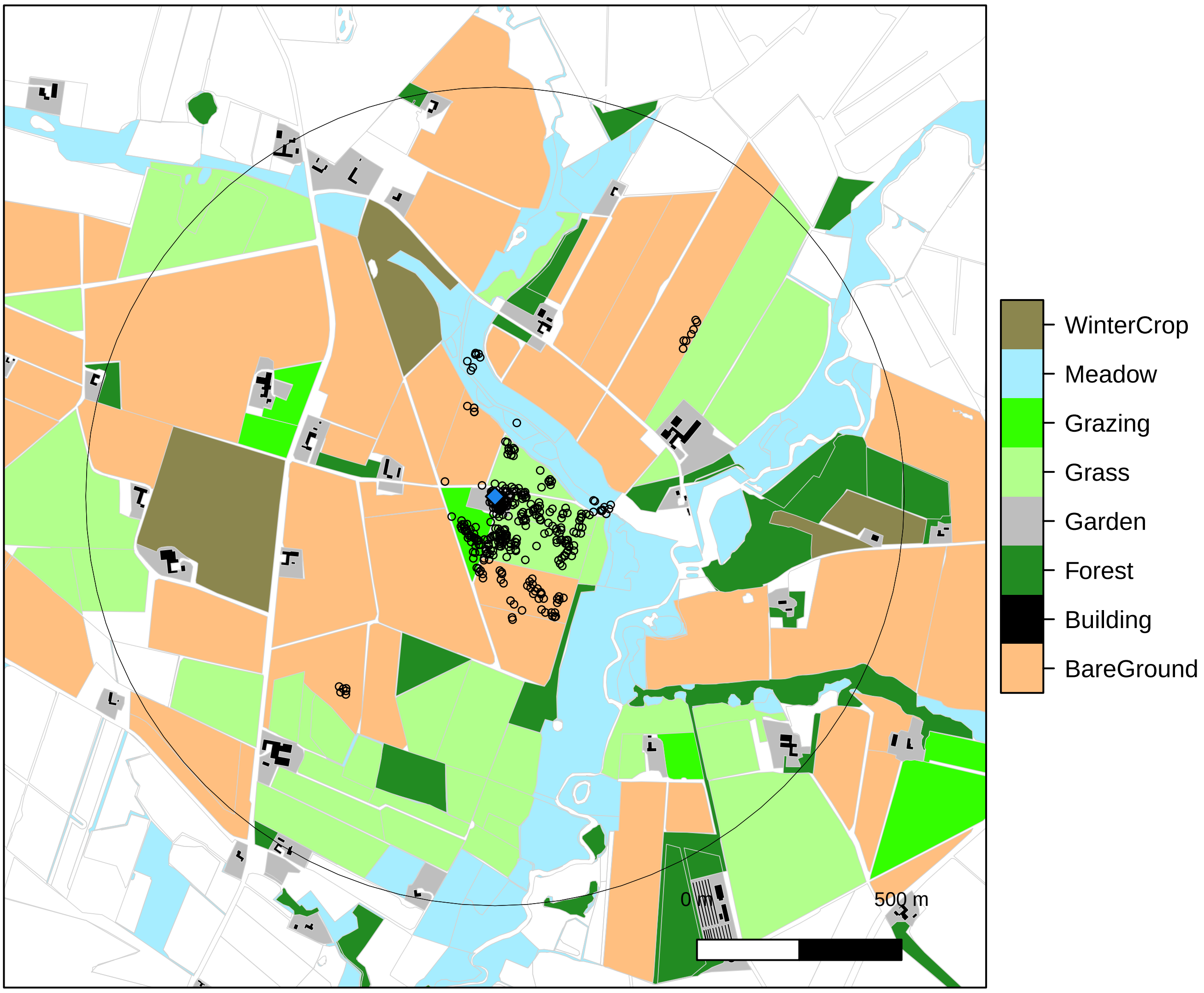

0 m
500 m
WinterCrop
Meadow
Grazing
Grass
Garden
Forest
Building
BareGround

S2−2015

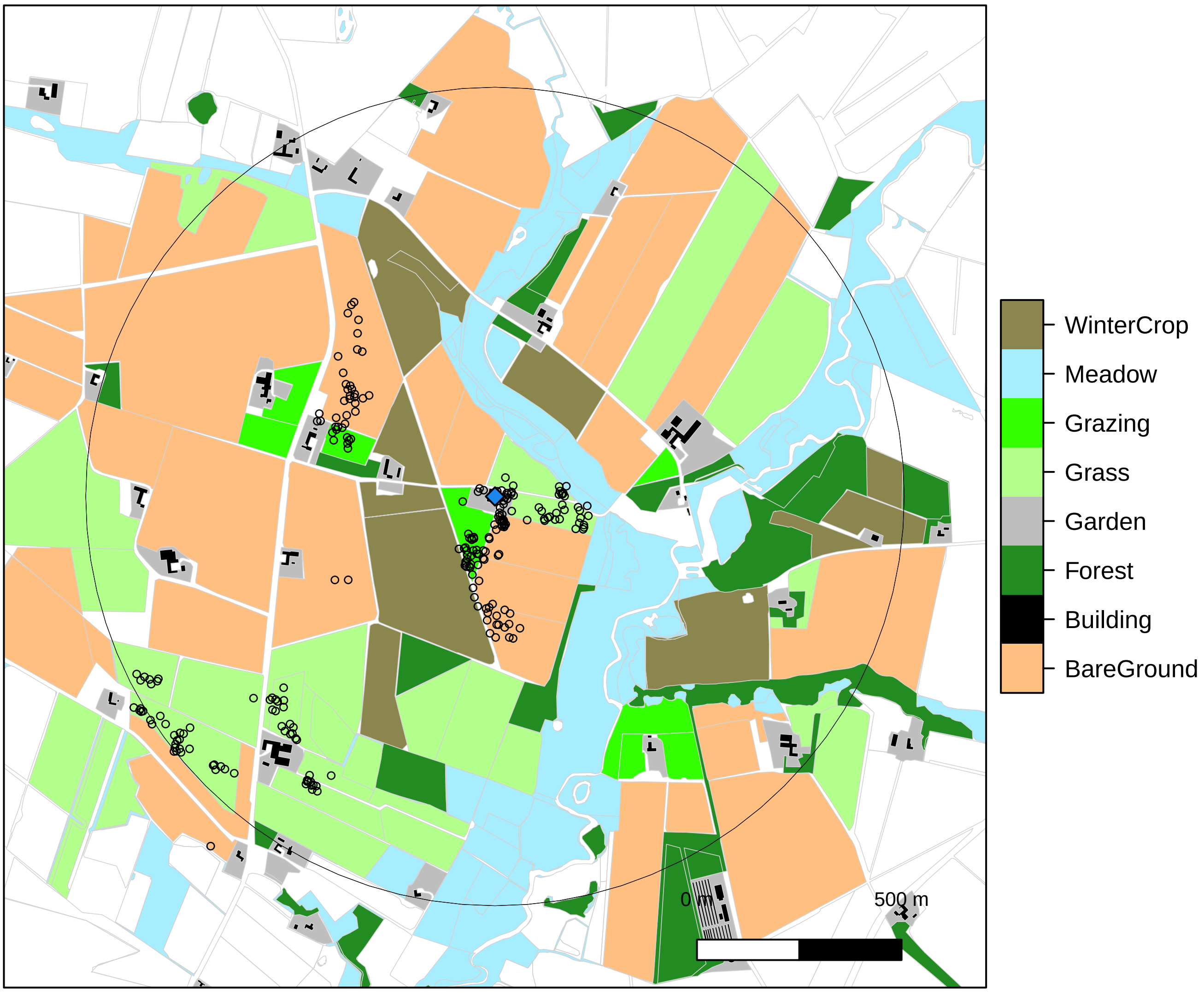

0 m
500 m
WinterCrop
Meadow
Grazing
Grass
Garden
Forest
Building
BareGround

S3−2015

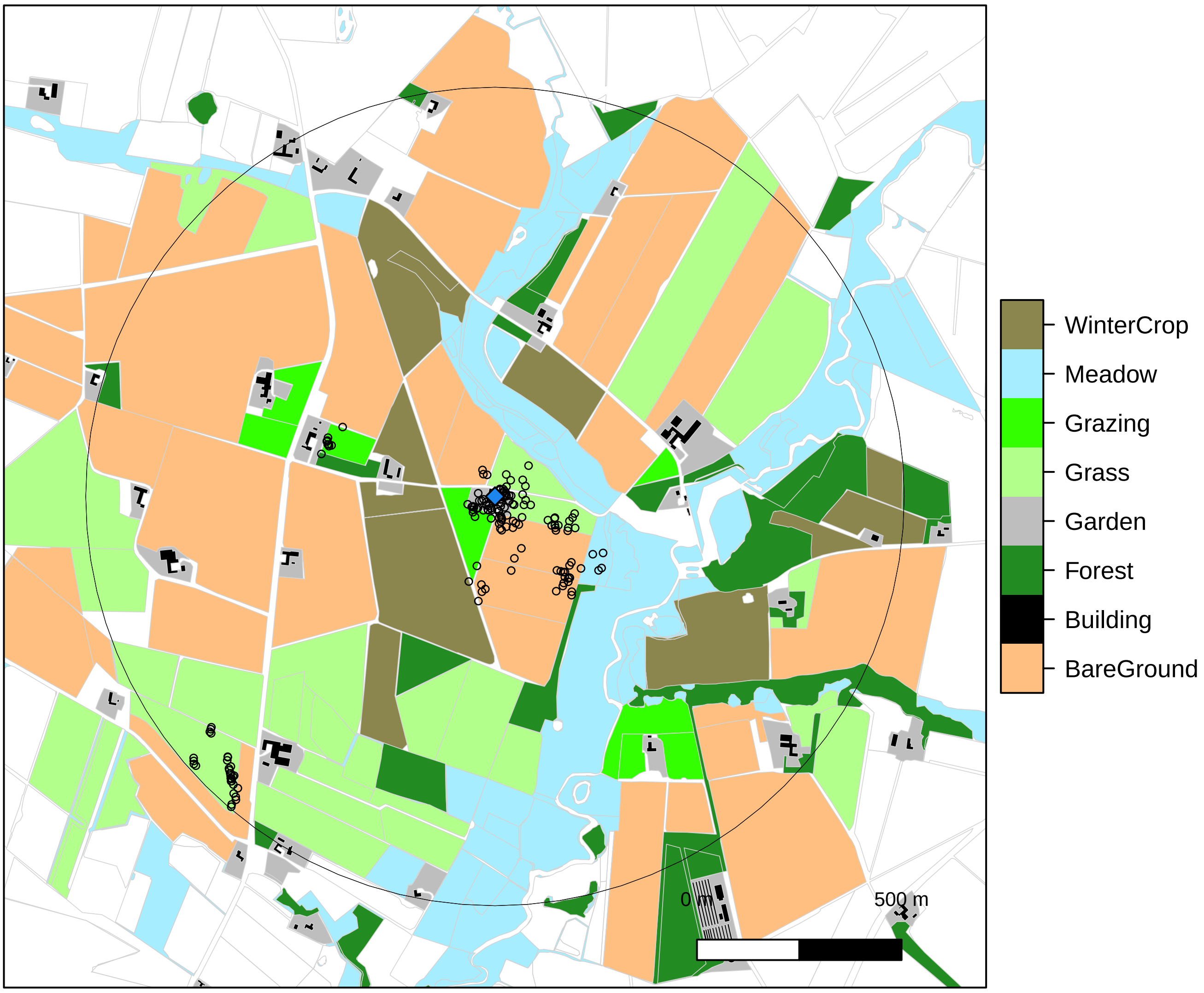

0 m
500 m
WinterCrop
Meadow
Grazing
Grass
Garden
Forest
Building
BareGround

S4a−2016

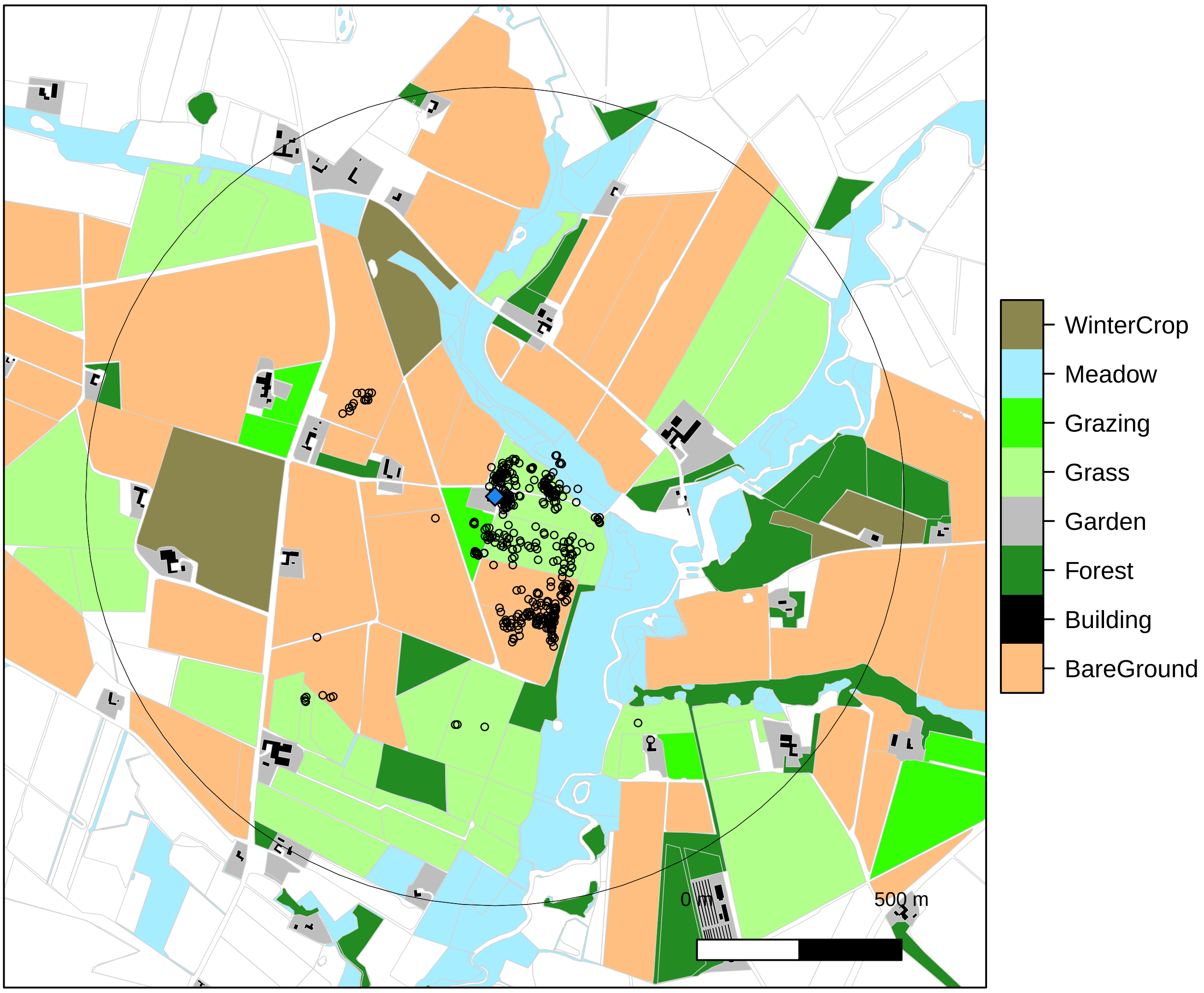
0 m
500 m
WinterCrop
Meadow
Grazing
Grass
Garden
Forest
Building
BareGround

S4b−2016

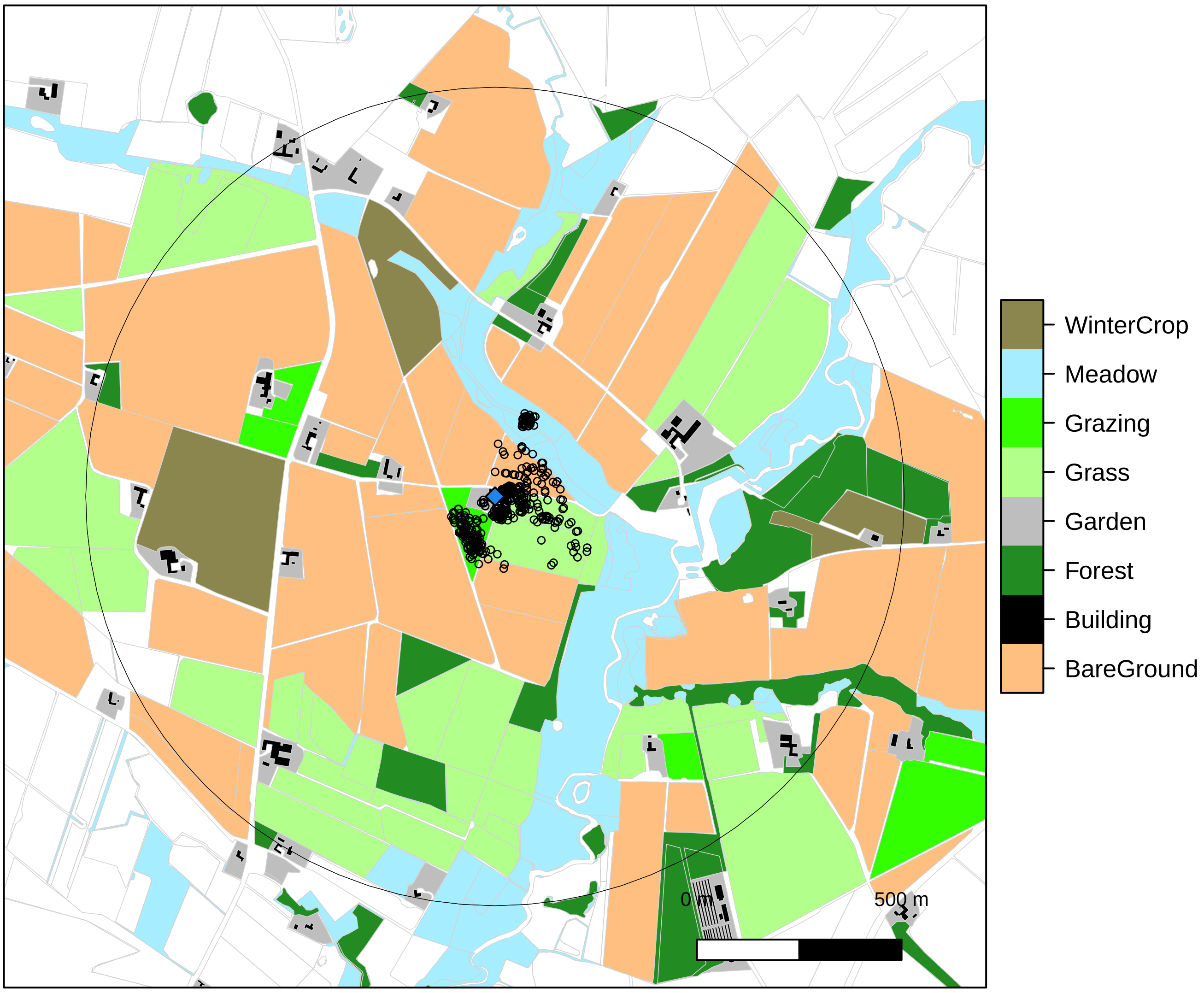
0 m
500 m
WinterCrop
Meadow
Grazing
Grass
Garden
Forest
Building
BareGround

S5−2015

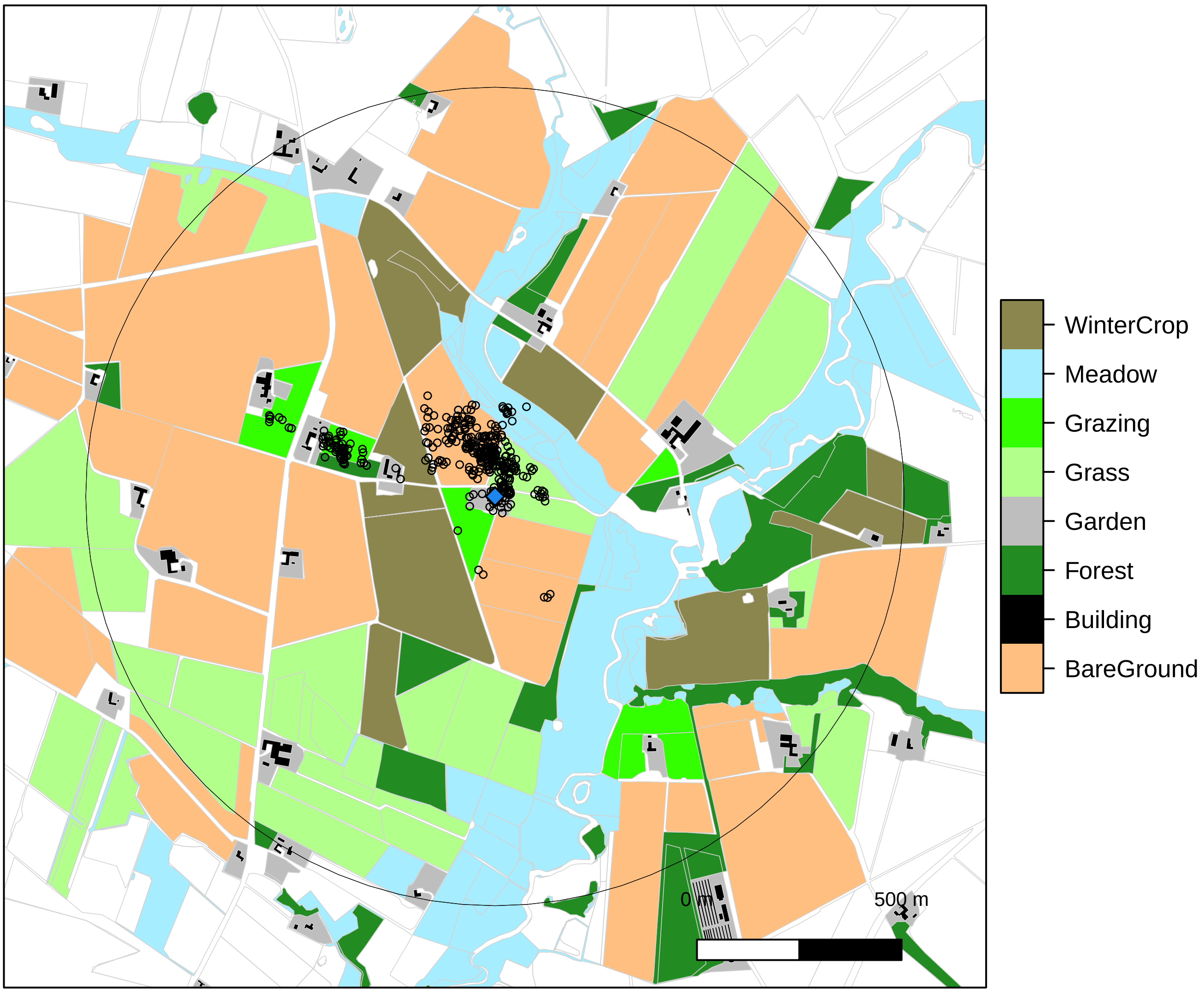

0 m
500 m
WinterCrop
Meadow
Grazing
Grass
Garden
Forest
Building
BareGround

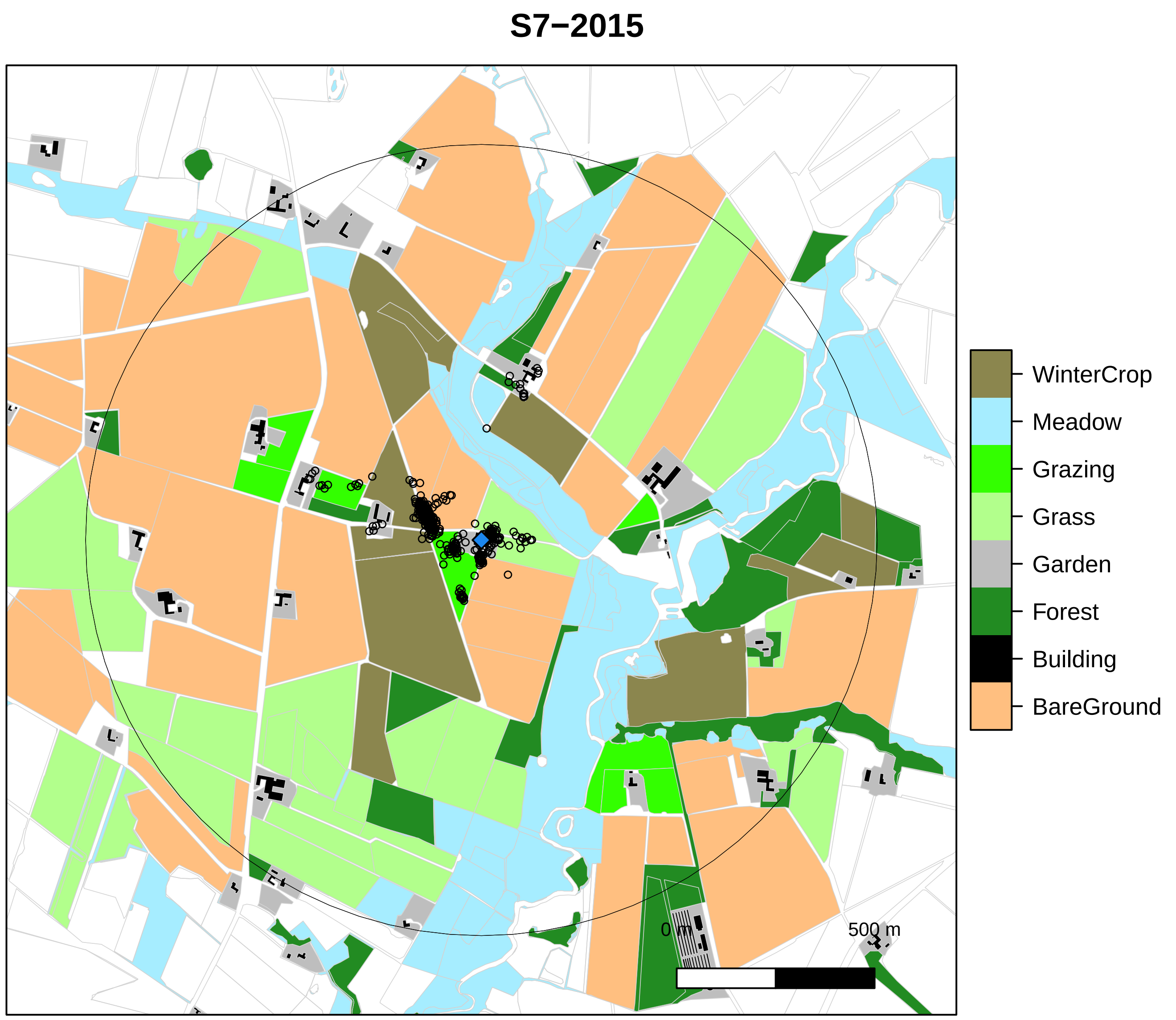
S7−2015
WinterCrop
Meadow
Grazing
Grass
Garden
Forest
Building
BareGround
0 m
500 m

S8−2015

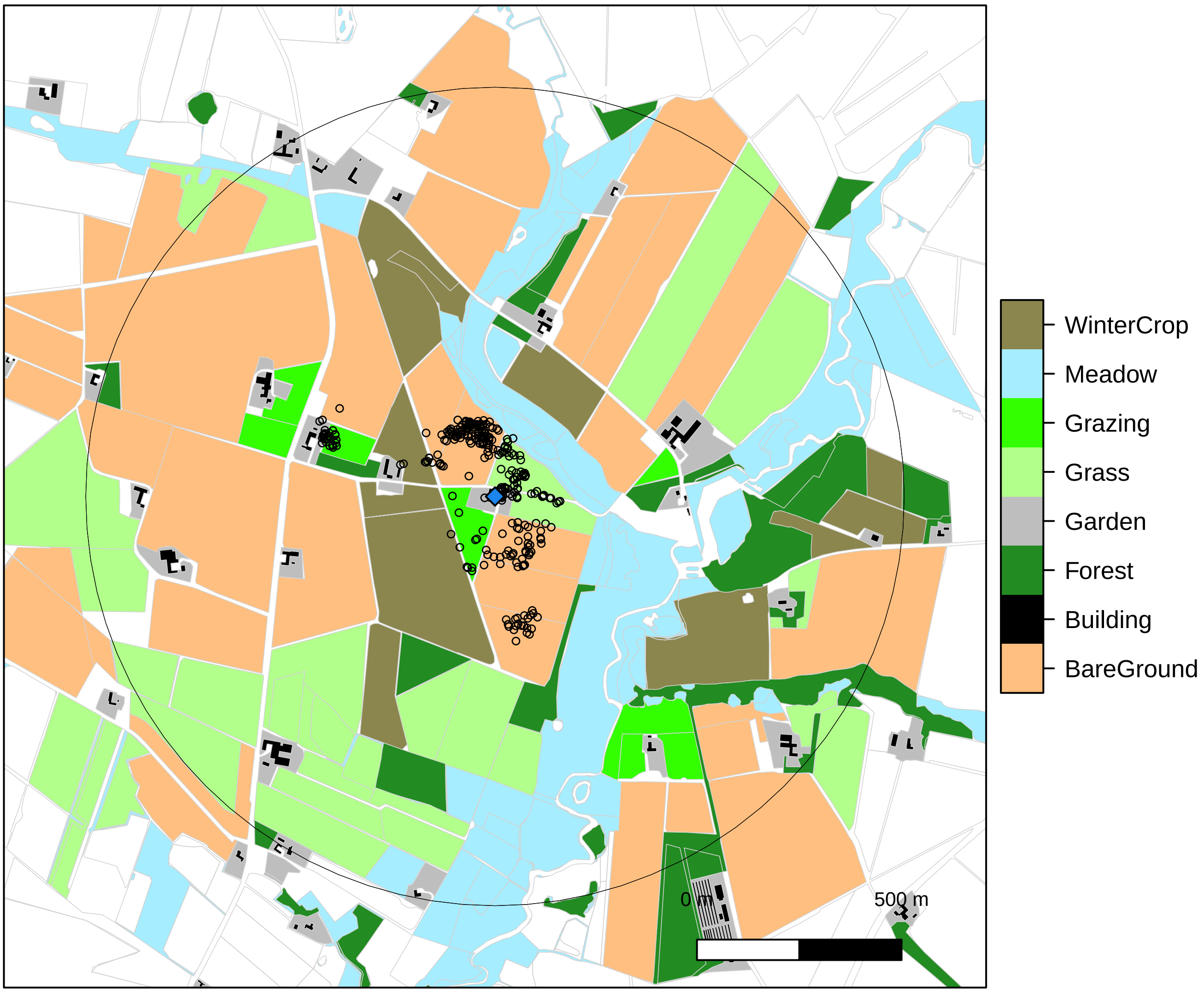

0 m
500 m
WinterCrop
Meadow
Grazing
Grass
Garden
Forest
Building
BareGround

S9−2015

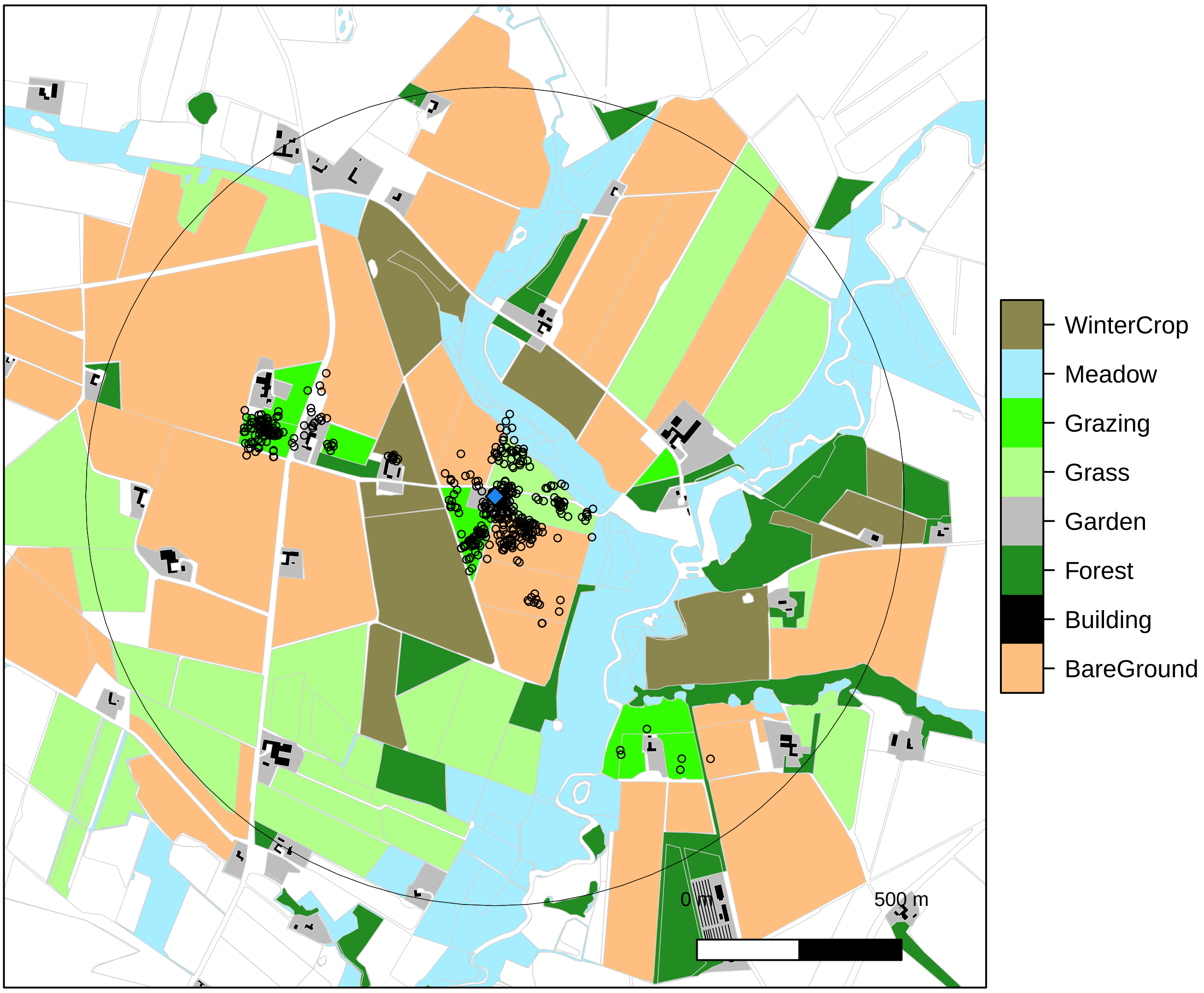

WinterCrop
Meadow
Grazing
Grass
Garden
Forest
Building
BareGround
0 m
500 m

S9a−2016

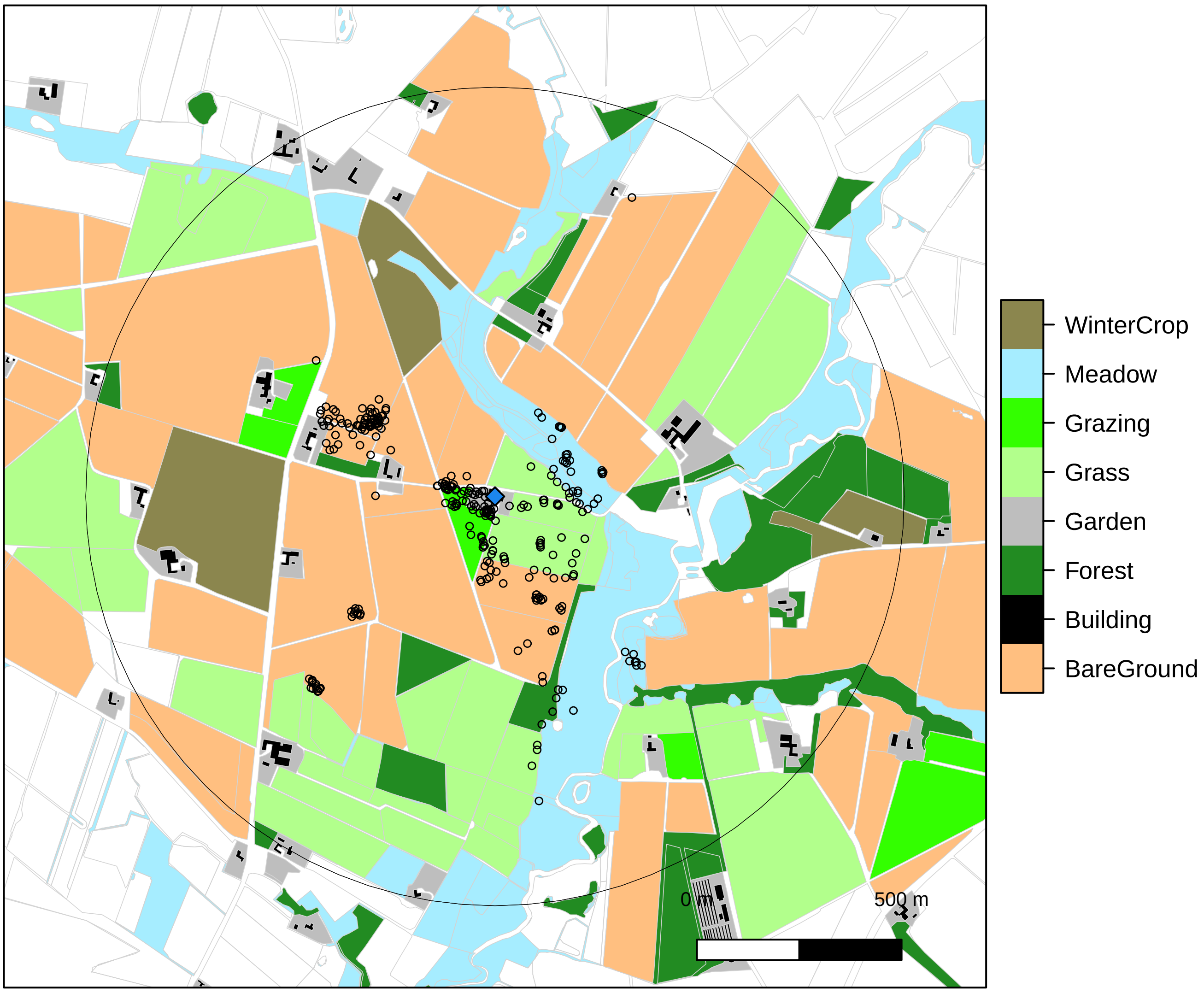
WinterCrop
Meadow
Grazing
Grass
Garden
Forest
Building
BareGround
0 m
500 m

S9b−2016

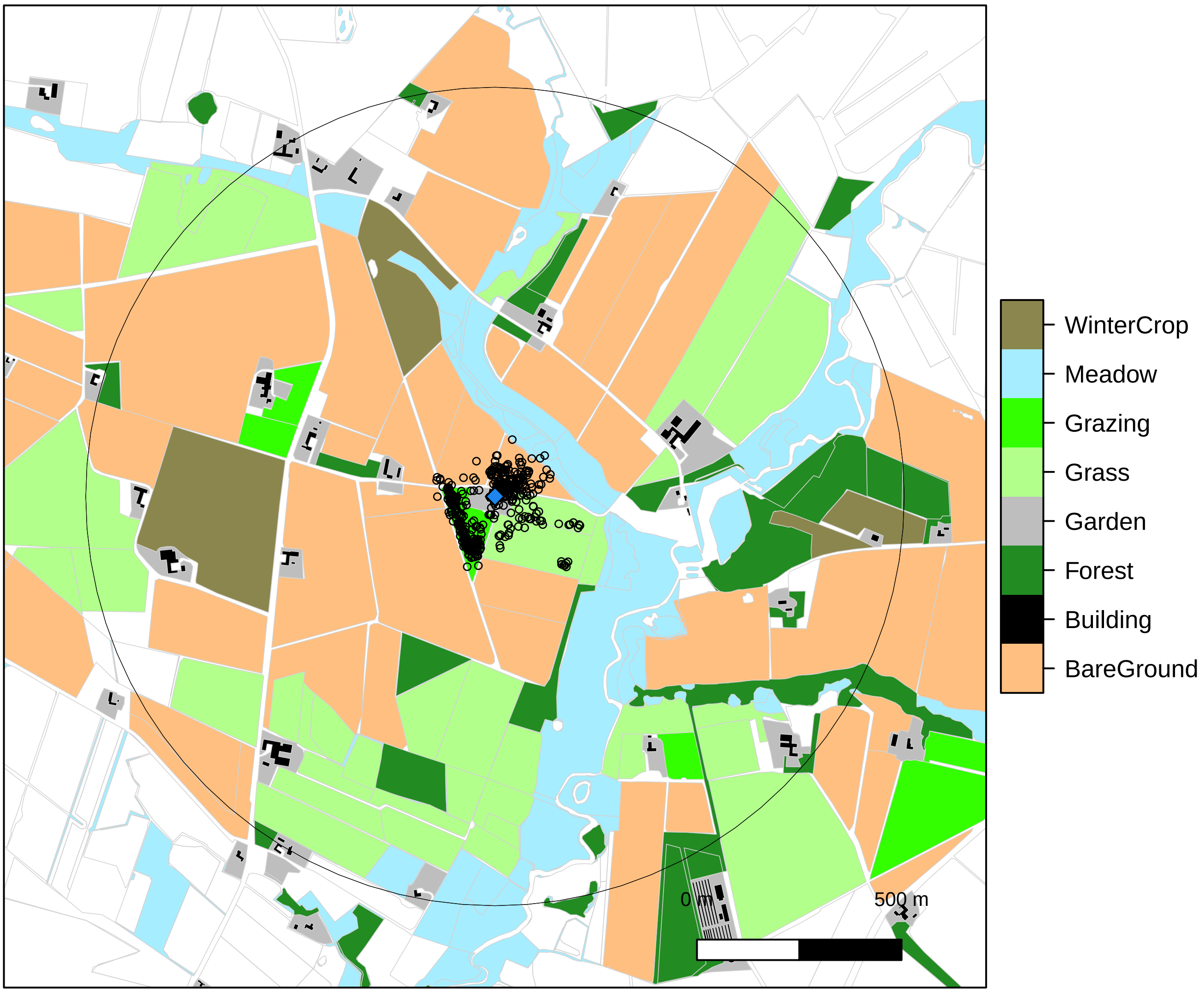
0 m
500 m
WinterCrop
Meadow
Grazing
Grass
Garden
Forest
Building
BareGround
