# Supplementary material for: Common Starlings (Sturnus vulgaris) increasingly select for grazed areas with increasing distance-to-nest
Source: PLoS One. 2017 Aug 3;12(8):e0182504. doi: 10.1371/journal.pone.0182504 (PMC5542446; doi:10.1371/journal.pone.0182504)
Supplement: S1 Table — (DOCX) [file pone.0182504.s003.docx]

Henning Heldbjerg, Anthony D. Fox, Peder V. Thellesen, Lars Dalby & Peter Sunde. Common Starlings (*Sturnus vulgaris)* increasingly select for grazed areas with increasing distance-to-nest
Plos One (2017)

(Short title: Distance dependent habitat selection among Common Starlings)

**Electronic supplementary material**

*Table S1. Information about the 17 loggers/Starlings showing year, logger, sex, start and length of each logger period, number of foraging positions and registered mean and max distance. Each logger provides a data file (txt) giving information on every fix until the battery runs short of energy. Each fix comprises the following information: Date (dd/mm/yyyy), Time (hh:mm:ss), Latitude, Longitude, Altitude, Instantaneous speed ground (km/h), Number of satellites, Horizontal Dilution of Precision (HDOP, a value which describes the geometric strength of satellite configuration on GPS accuracy; lower values indicate higher accuracy) and GSV Value (a measure of satellite reception power, higher values indicating better satellite reception; see [17].*
